# Supplementary figures and images for: α-Hemolysin promotes uropathogenic E. coli persistence in bladder epithelial cells via abrogating bacteria-harboring lysosome acidification
Source: PLoS Pathog. 2023 May 11;19(5):e1011388. doi: 10.1371/journal.ppat.1011388 (PMC10204954; doi:10.1371/journal.ppat.1011388)

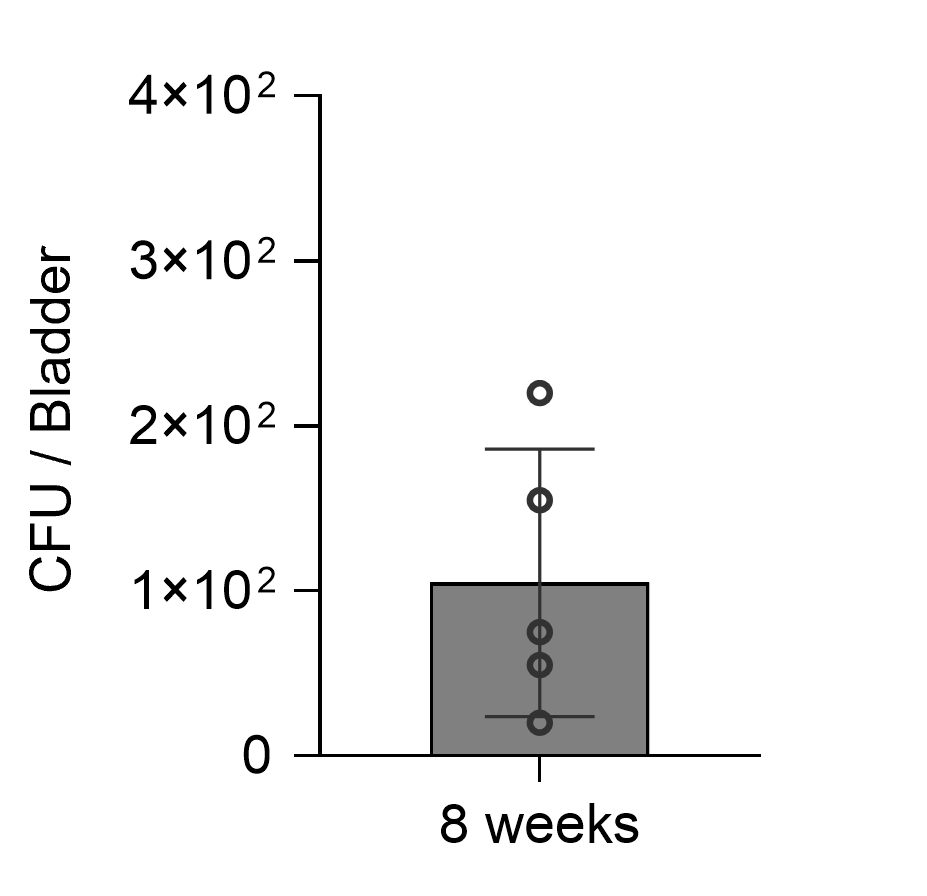

Supplement: S1 Fig — C57BL/6 female mice were infected by intravesical instillation of the UPEC CI5 strain and bacterial CFUs in the infected bladders were measured at 8 weeks post-infection. (TIF) [file ppat.1011388.s001.tif]

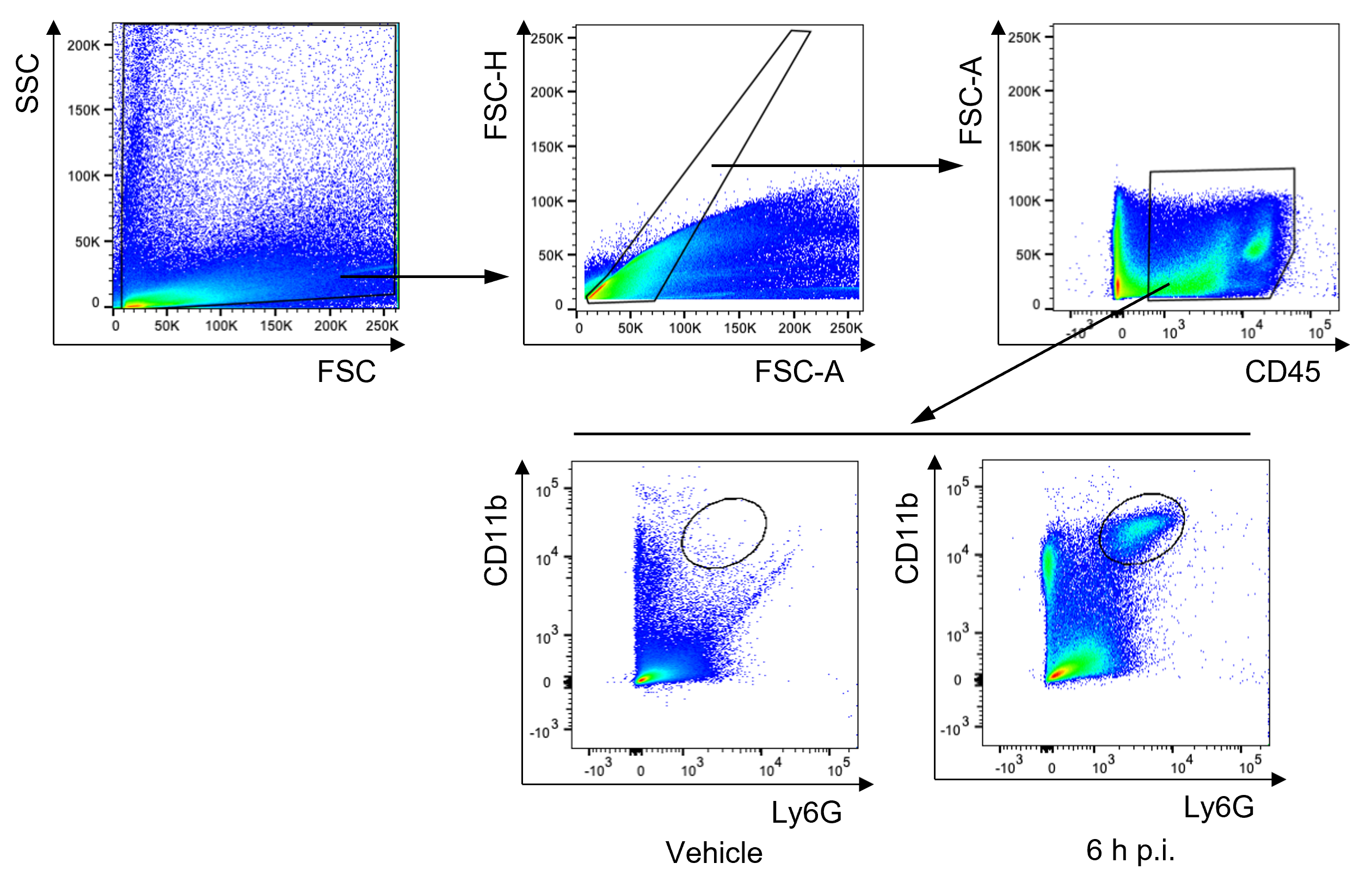

Supplement: S2 Fig — Ly6G+ CD11b+ CD45+ cells in the mouse bladders were counted by flow cytometry. (TIF) [file ppat.1011388.s002.tif]

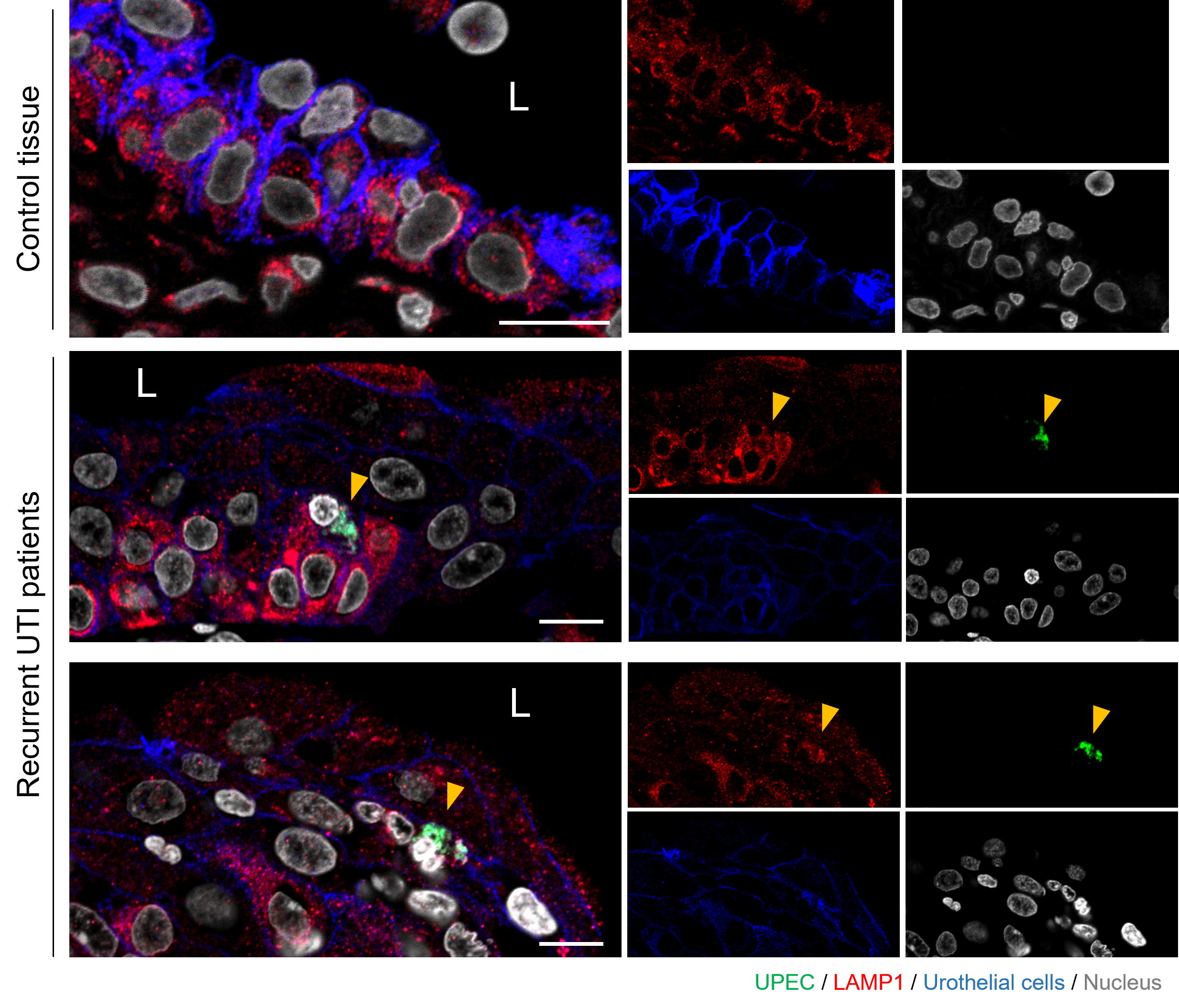

Supplement: S3 Fig — Bladder biopsies were obtained from recurrent UTI patients or patients with no history of UTI in past two year. These tissues were immunostained for UPEC (E. coli, green), urothelium (E-cadherin, blue), lysosomal vesicle (LAMP1, red), and nucleus (DAPI, gray). Yellow arrowheads indicate UPEC. “L”: lumen of the bladder. Scale bars: 10 μm. (TIF) [file ppat.1011388.s003.tif]

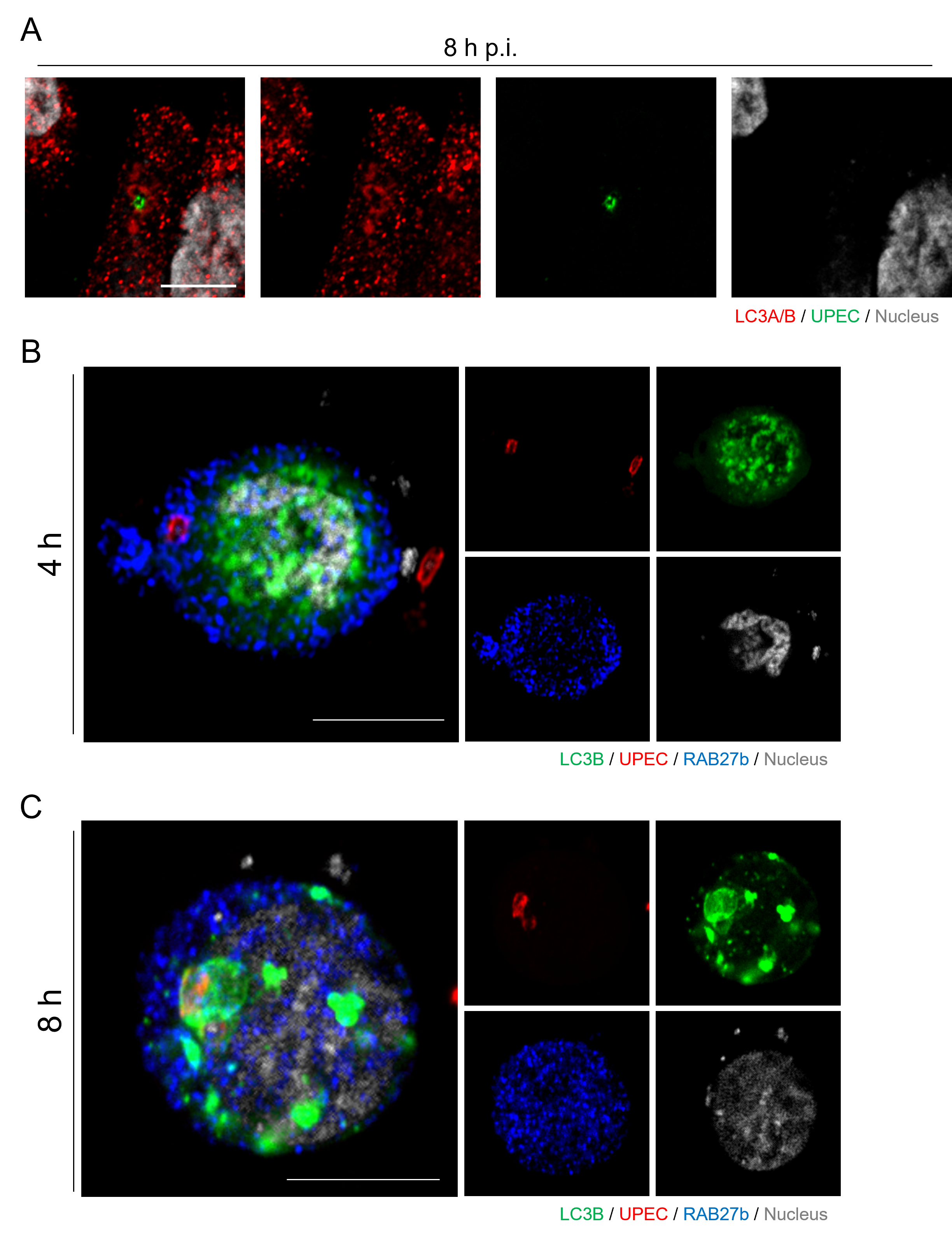

Supplement: S4 Fig — (A) Human 5637 BECs were infected with UPEC CI5 strain for 8 h and were stained with anti-LC3A/B (red) and anti-UPEC (green) antibodies. (B, C) Human 5637 BECs were transfected with pEGFP-LC3 (green) to specifically trace LC3 autophagy component. Next day, the cells were infected with UPEC CI5 strain. After 4 or 8 h p.i., cells were stained with anti-E. coli (red) and anti-RAB27b (blue) antibodies. Representative images are from randomly selected regions. Scale bars: 10 μm. (TIF) [file ppat.1011388.s004.tif]

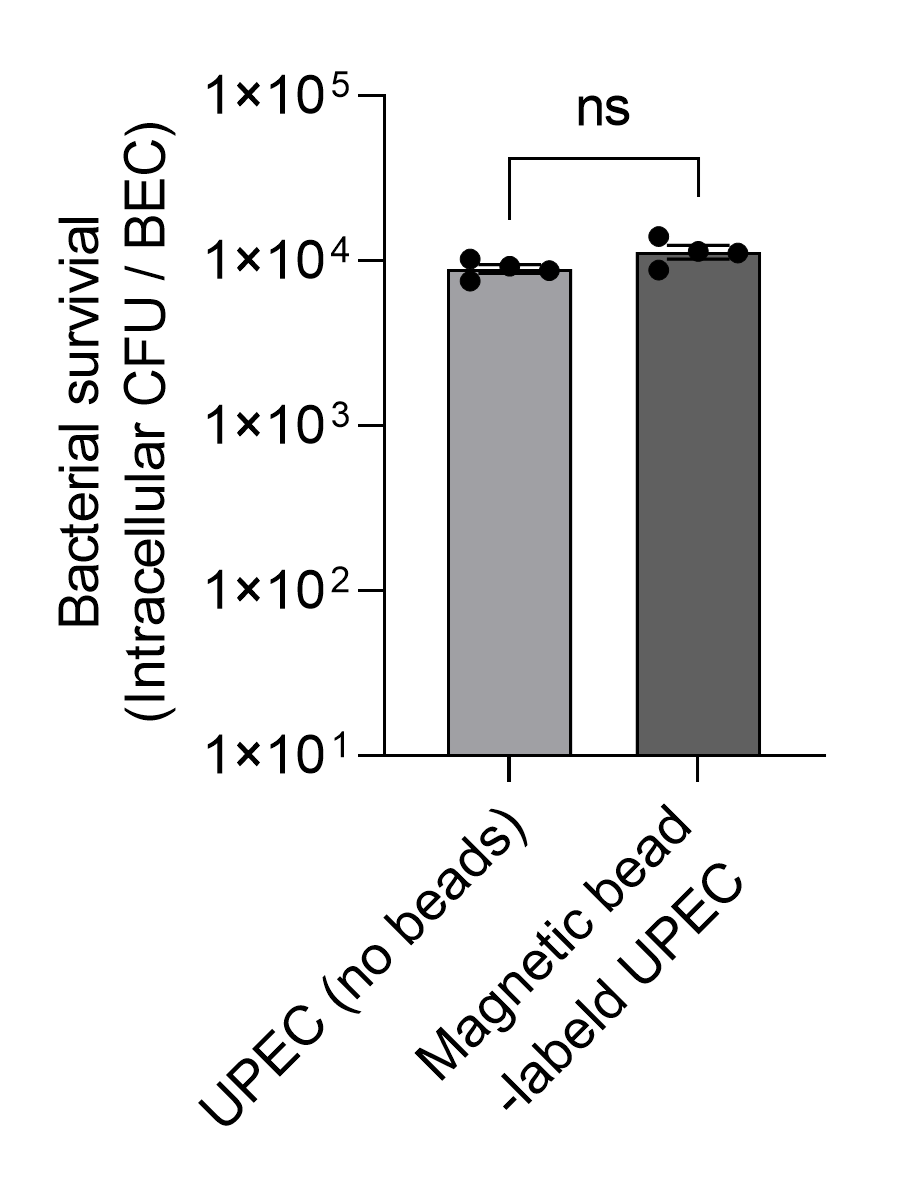

Supplement: S5 Fig — Human 5637 BECs were treated with magnetic bead-labeled UPEC CI5 strain or UPEC CI5 strain (no labeled beads). After 2 h of incubation with gentamicin to remove extracellular UPEC, intracellular CFU were examined. ns: not significant. (TIF) [file ppat.1011388.s005.tif]

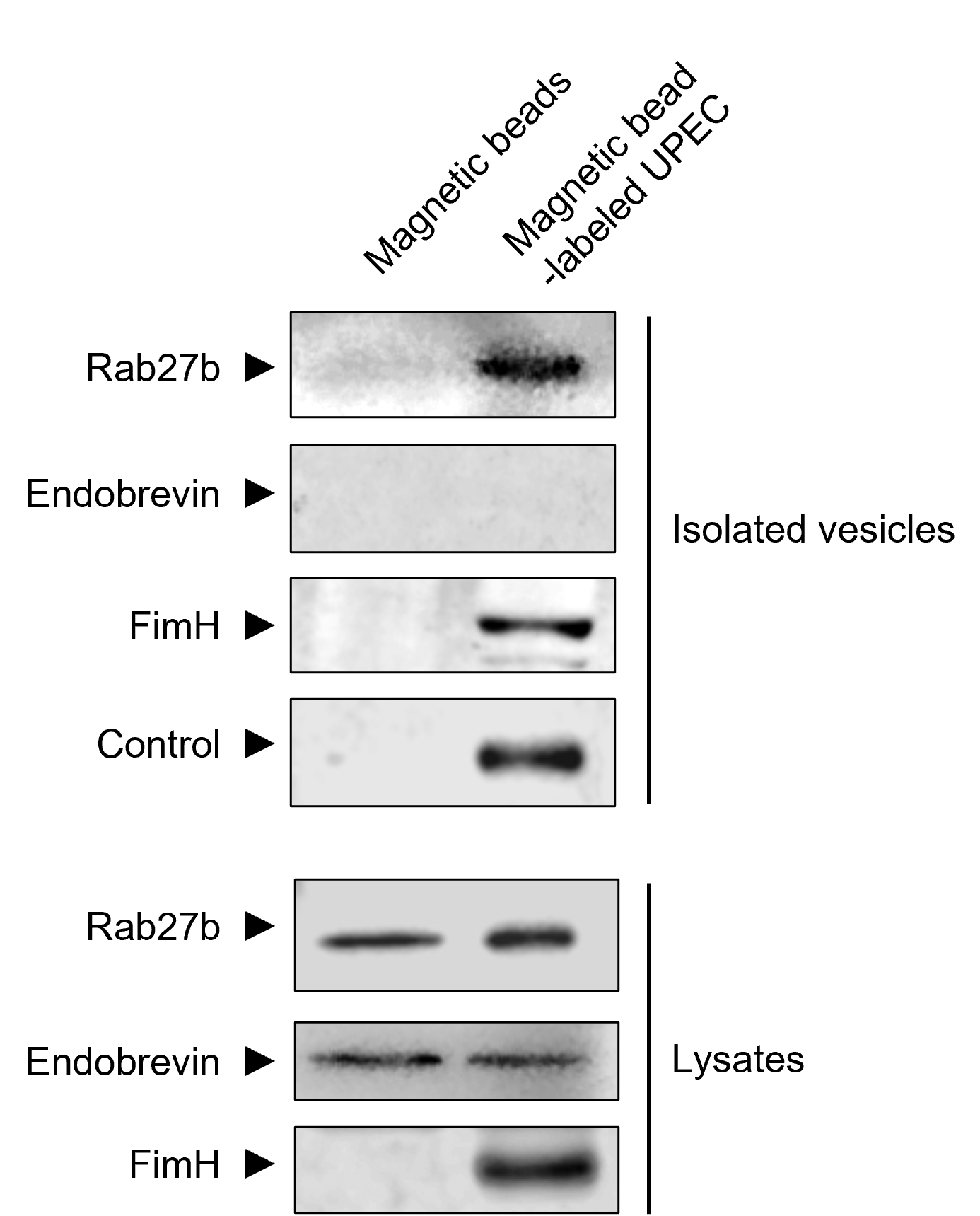

Supplement: S6 Fig — Human 5637 BECs were incubated with magnetic beads alone or with magnetic bead-labeled UPEC. After two hours of incubation, isolated beads or cell lysates from each condition were analyzed for the expression of RAB27b, Endobrevin/VAMP8, and FimH. Gapdh was used for loading control of UPEC-containing vesicle. (TIF) [file ppat.1011388.s006.tif]

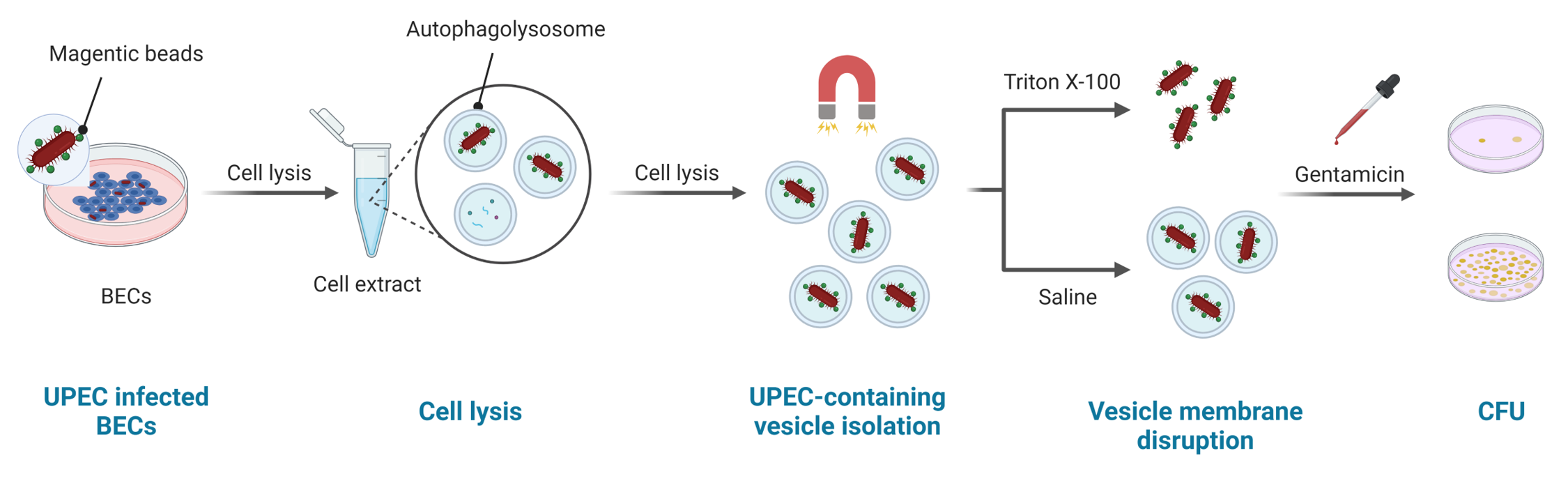

Supplement: S7 Fig — Human 5637 BECs were seeded and infected with magnetically labeled UPECs. After lysing cells by passing the cells through needles, UPEC-containing vesicles were isolated with magnet. Isolated vesicles containing UPECs were treated with 0.1% Triton X-100 or saline, then incubated with gentamicin before plating on McConkey agar plates. (TIF) [file ppat.1011388.s007.tif]

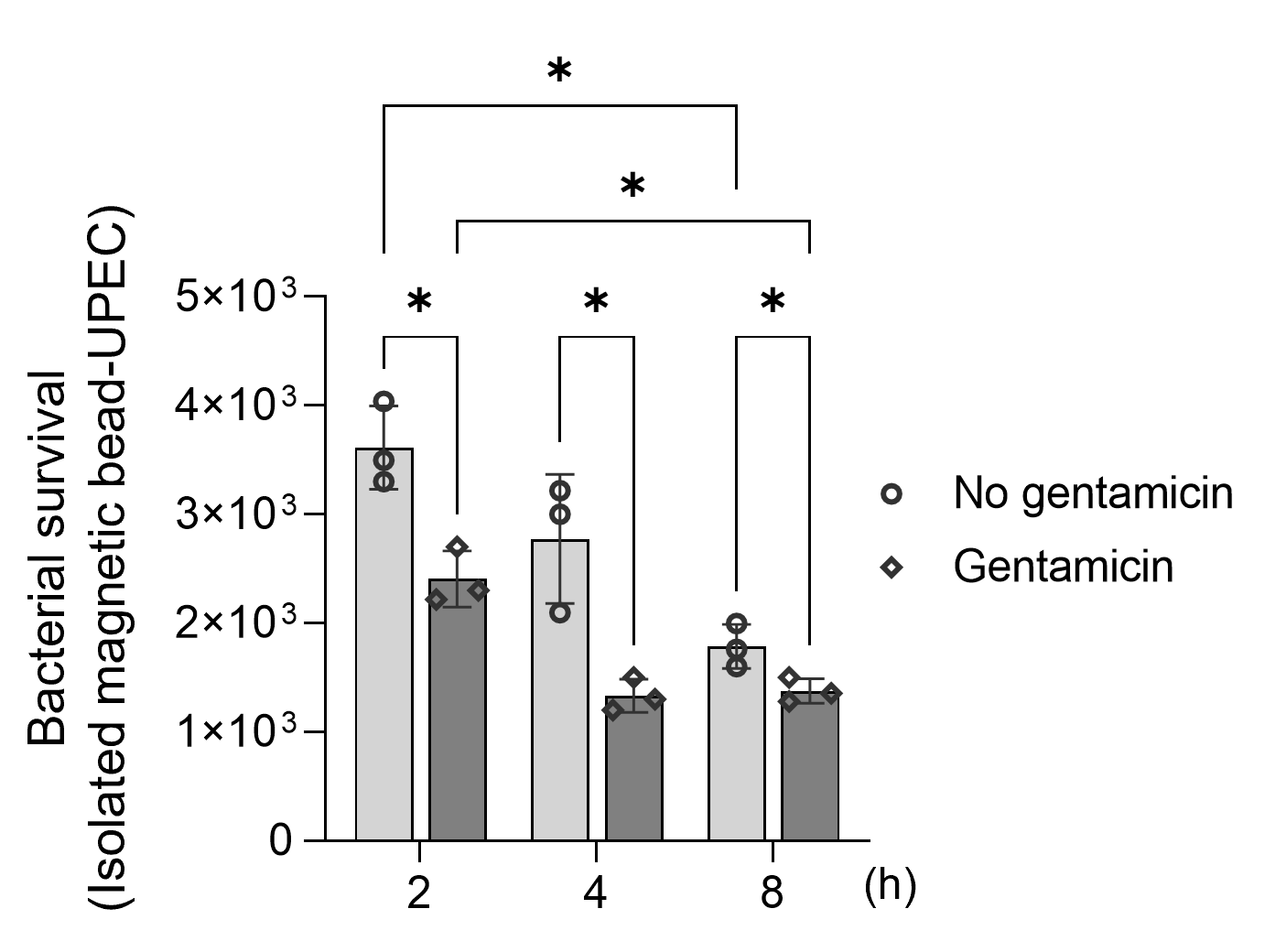

Supplement: S8 Fig — Data were analyzed by two-way ANOVA. *P<0.05 (TIF) [file ppat.1011388.s008.tif]

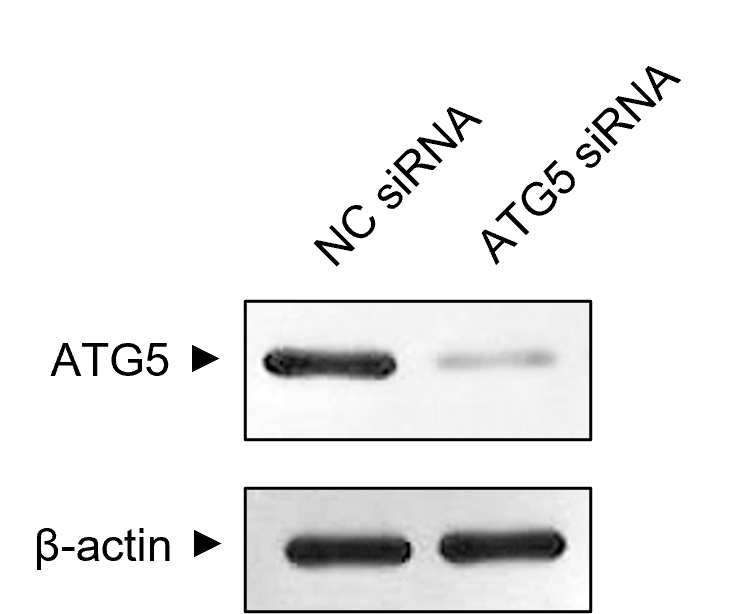

Supplement: S9 Fig — The 5637 BECs transfected with NS siRNA or ATG5 siRNA and immunoblotted using an anti-ATG5 antibody to examine the expression of ATG5. Anti-β-actin antibody was used for a loading control. (TIF) [file ppat.1011388.s009.tif]

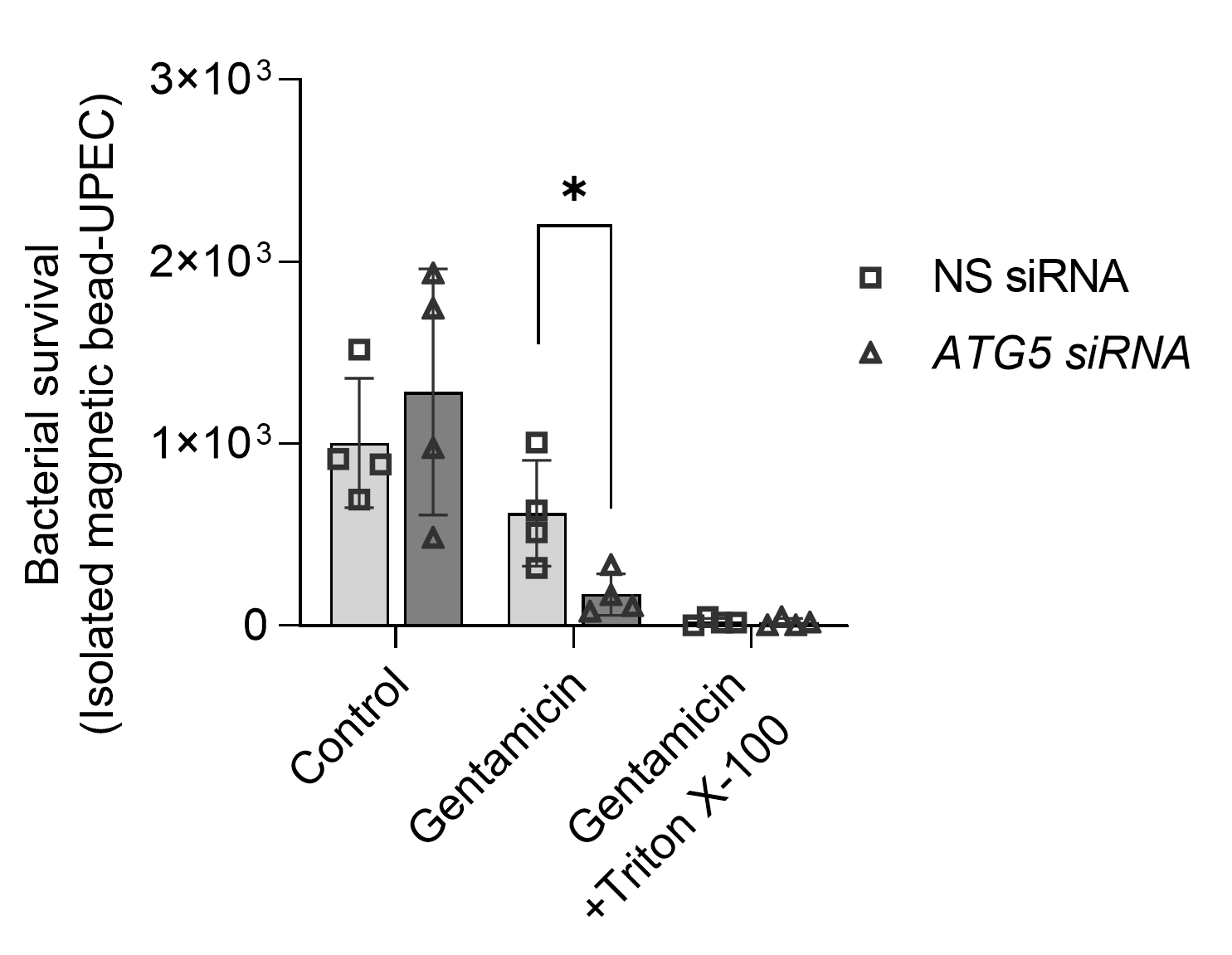

Supplement: S10 Fig — Data were analyzed by two-way ANOVA. *P<0.05 (TIF) [file ppat.1011388.s010.tif]

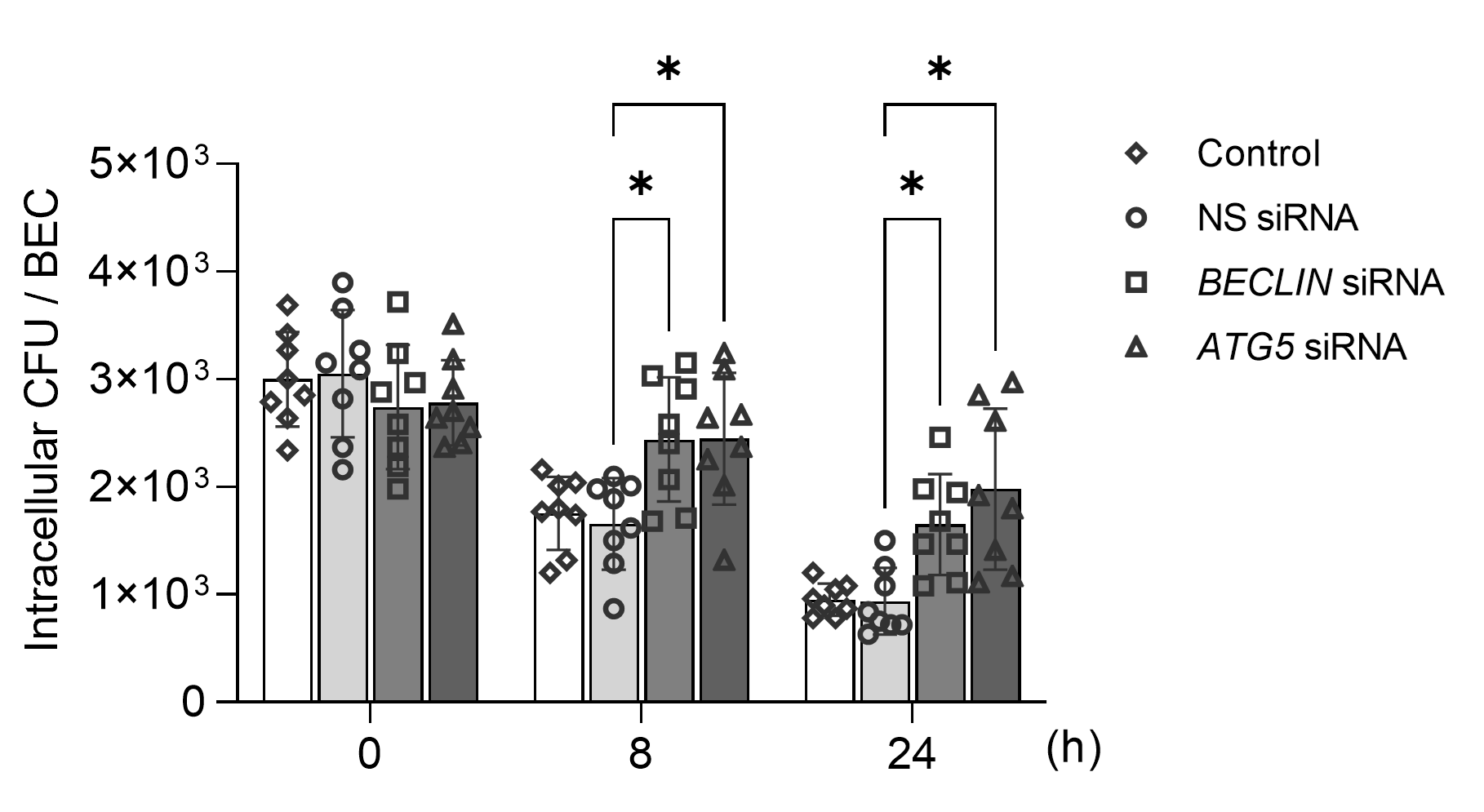

Supplement: S11 Fig — Data were analyzed by one-way ANOVA. *P<0.05 (TIF) [file ppat.1011388.s011.tif]

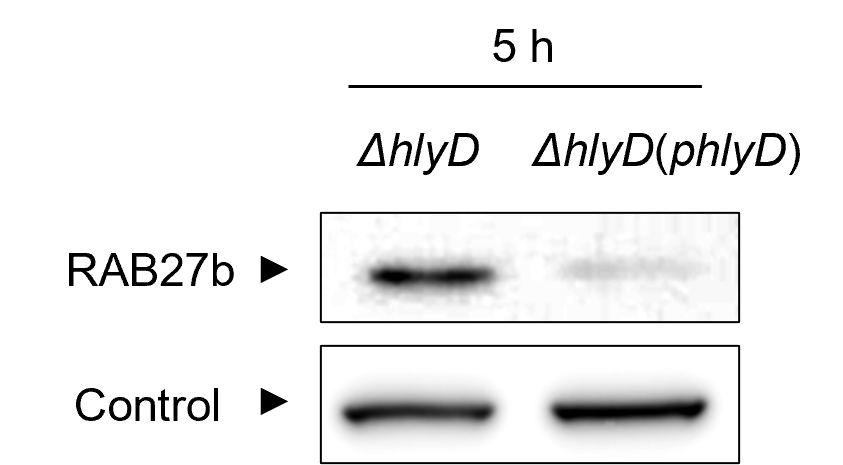

Supplement: S12 Fig — ΔhlyD UPEC strain was transformed with phlyD plasmid to generate ΔhlyD(phlyD) strain. Human 5637 BECs were infected with magnetic beads labeled ΔhlyD(phlyD) strain or ΔhlyD with empty plasmid. After 5 h of infection, isolated UPEC-containing vesicles were analyzed for the expression of RAB27b. Gapdh was used for loading control of UPEC-containing vesicles. (TIF) [file ppat.1011388.s012.tif]

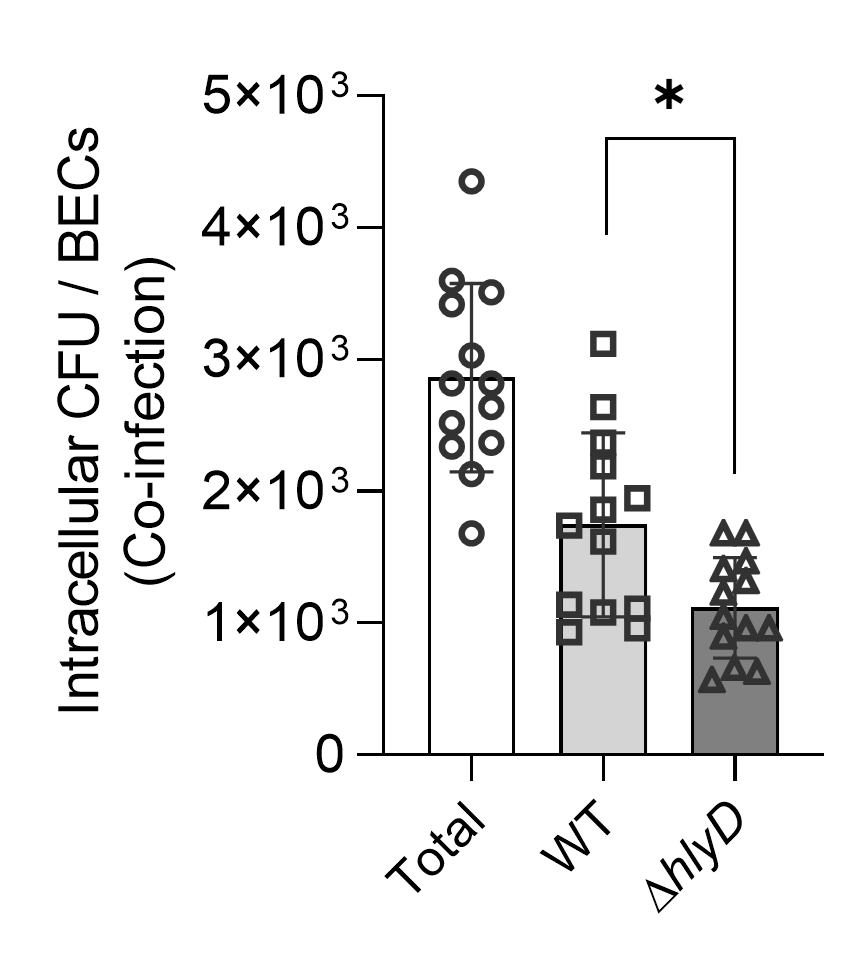

Supplement: S13 Fig — Data were analyzed by one-way ANOVA. *P<0.05 (TIF) [file ppat.1011388.s013.tif]

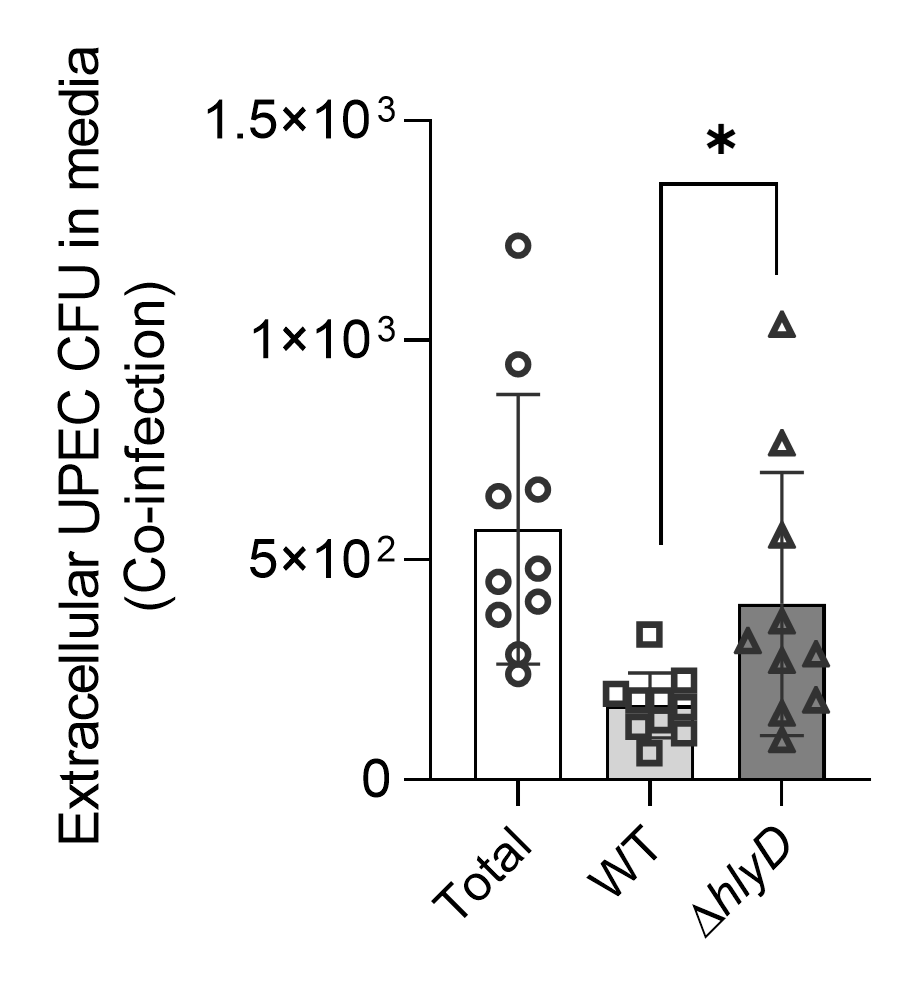

Supplement: S14 Fig — Data were analyzed by one-way ANOVA. *P<0.05 (TIF) [file ppat.1011388.s014.tif]

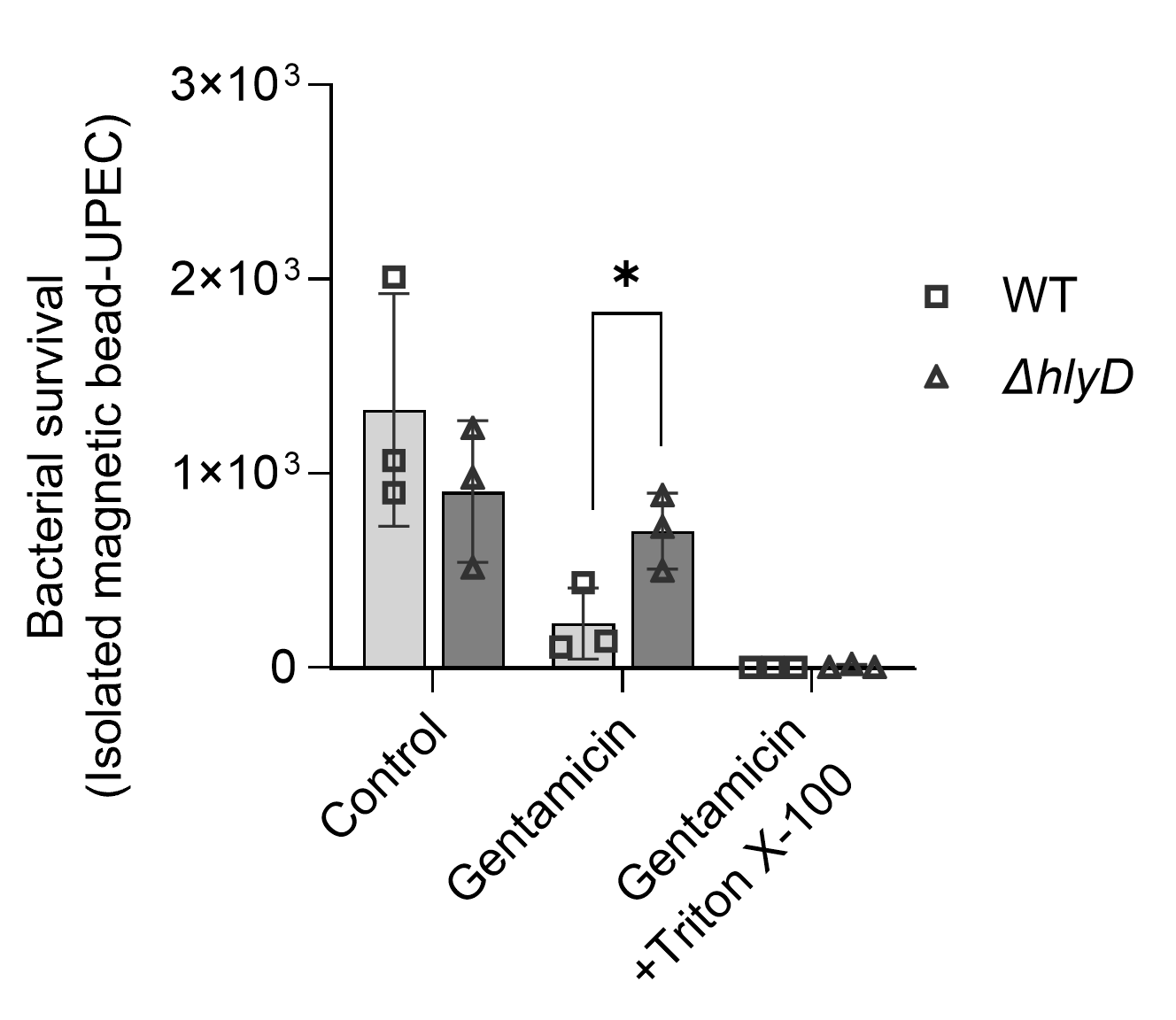

Supplement: S15 Fig — Data were analyzed by two-way ANOVA. *P<0.05 (TIF) [file ppat.1011388.s015.tif]

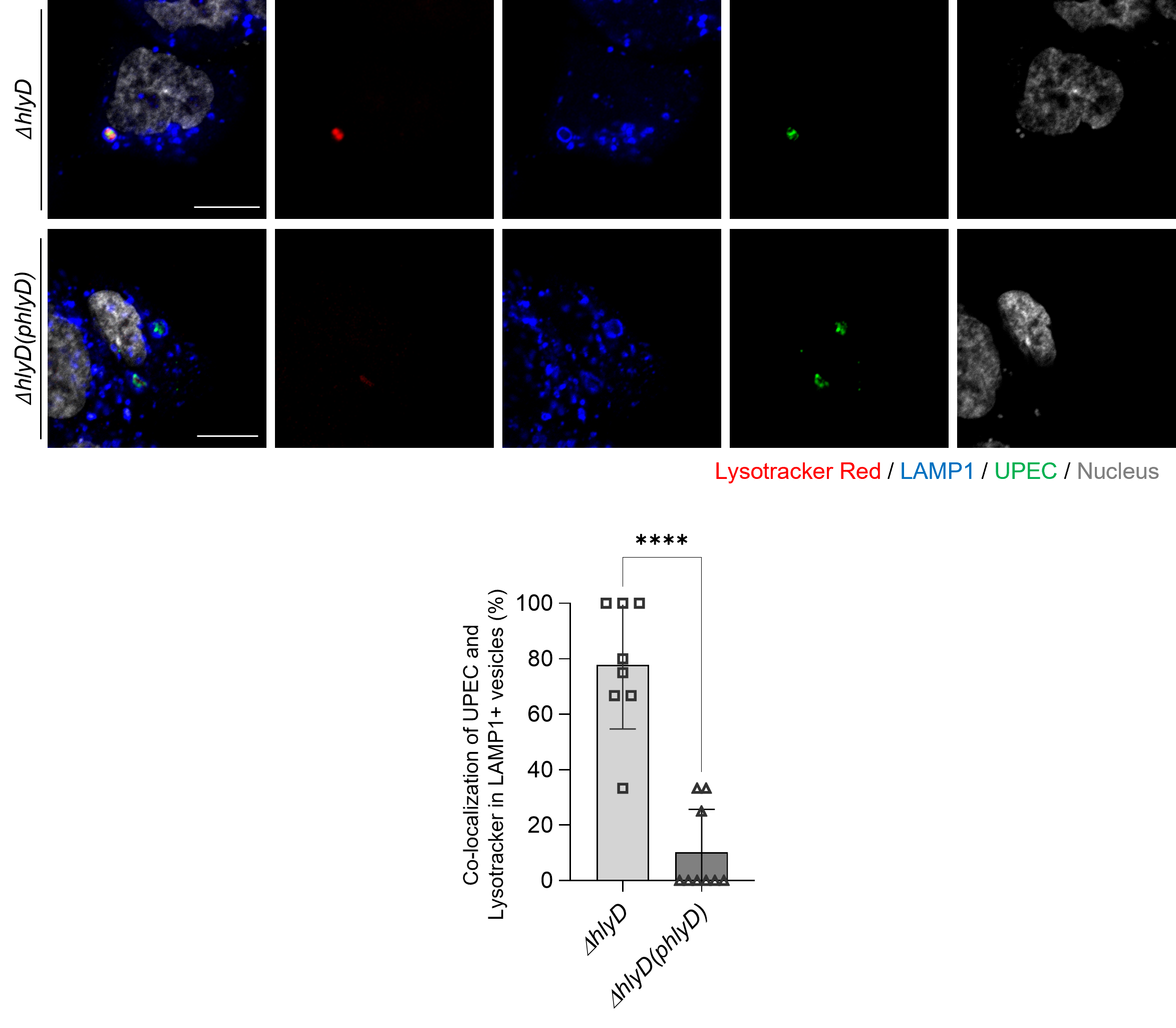

Supplement: S16 Fig — ΔhlyD UPEC strain was transformed with phlyD plasmid to generate ΔhlyD(phlyD) strain. Human 5637 BECs were infected with ΔhlyD(phlyD) strain or ΔhlyD with empty plasmid. After 24 h of infection, BECs were dyed with LysoTracker (red) to trace the acidification of UPEC-containing vesicles. Anti-UPEC (green) and anti-LAMP1 (blue) antibodies were used to stain the fixed cells. Quantitative data from two independent experiments were analyzed. Data are shown as mean ±SD. Data were analyzed by unpaired two-tailed Student’s t-test. ****P<0.0001. Scale bar: 10 μm. (TIF) [file ppat.1011388.s016.tif]

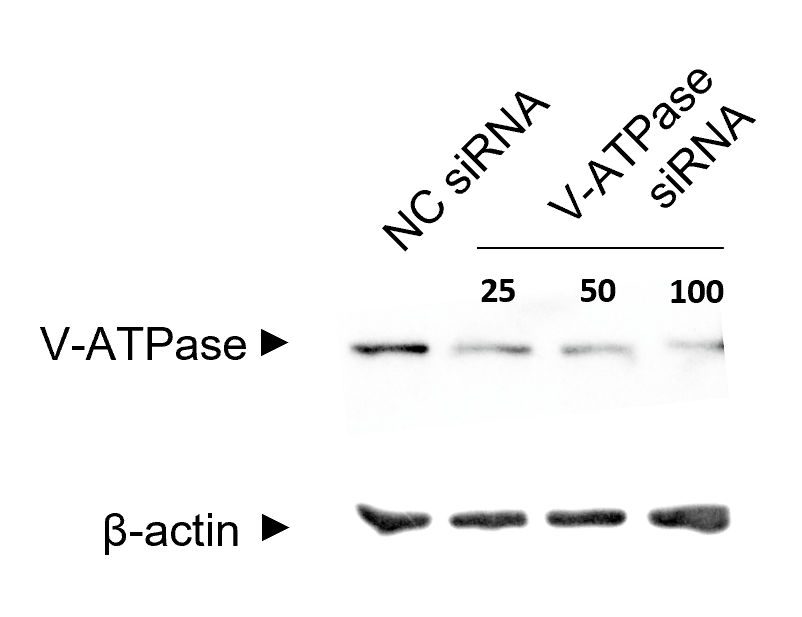

Supplement: S17 Fig — The 5637 BECs transfected with NS siRNA or V-ATPase siRNA (25, 50, or 100 pmol) and immunoblotted using an anti-V-ATPase antibody to examine the expression of V-ATPase. Anti-β-actin antibody was used for a loading control. (TIF) [file ppat.1011388.s017.tif]

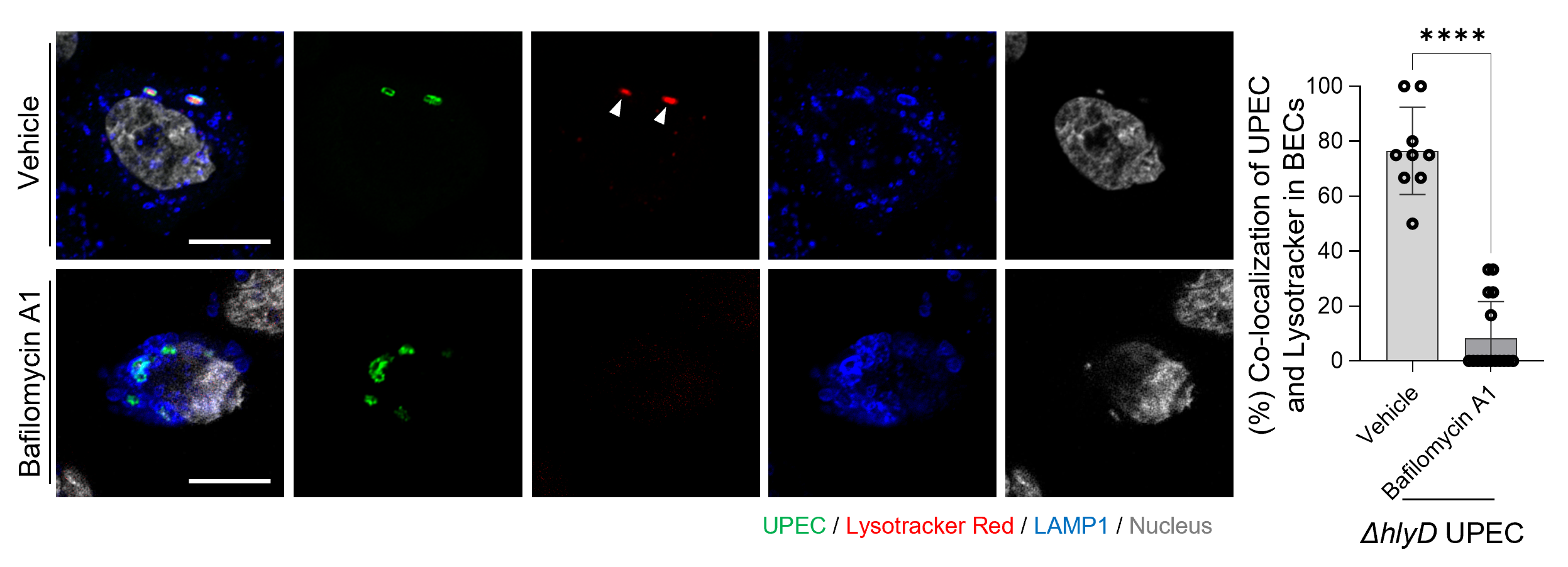

Supplement: S18 Fig — Human 5637 BECs were infected with UPEC ΔhlyD strain, and extracellular UPEC was removed by gentamycin treatment. Then, infected BECs were incubated in media containing Bafilomycin A1, a known pharmacological inhibitor of V-ATPase (vacuolar H+-ATPase), at 100 nM concentration. After 24 h post-treatment, LysoTracker Red dye was applied and then stained with anti-LAMP1 (blue) or anti-UPEC (green) antibodies for confocal microscopy imaging. The number of UPEC co-localized with LysoTracker Red dye was quantified in randomly chosen fields. Data were analyzed by unpaired two-tailed Student’s t-test. ****P<0.0001. Scale bar: 10 μm. (TIF) [file ppat.1011388.s018.tif]

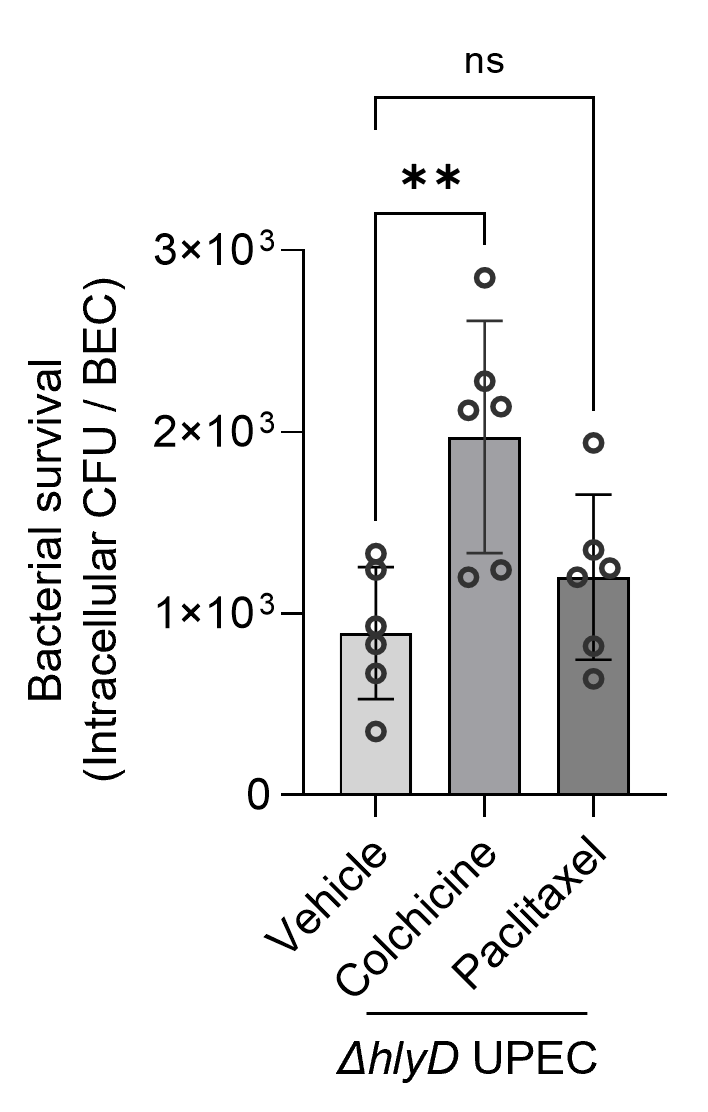

Supplement: S19 Fig — Data were analyzed by an ordinary one-way ANOVA (B, D, E, F). **P<0.01; n.s, not significant. (TIF) [file ppat.1011388.s019.tif]

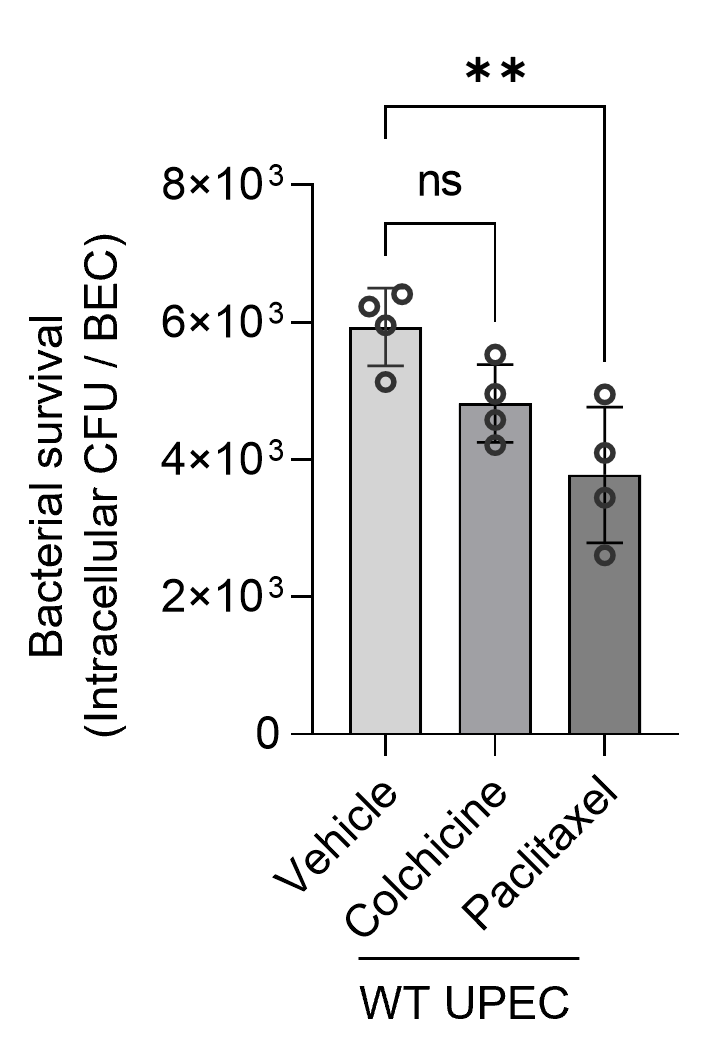

Supplement: S20 Fig — Data were analyzed by an ordinary one-way ANOVA. **P<0.01; n.s, not significant. (TIF) [file ppat.1011388.s020.tif]

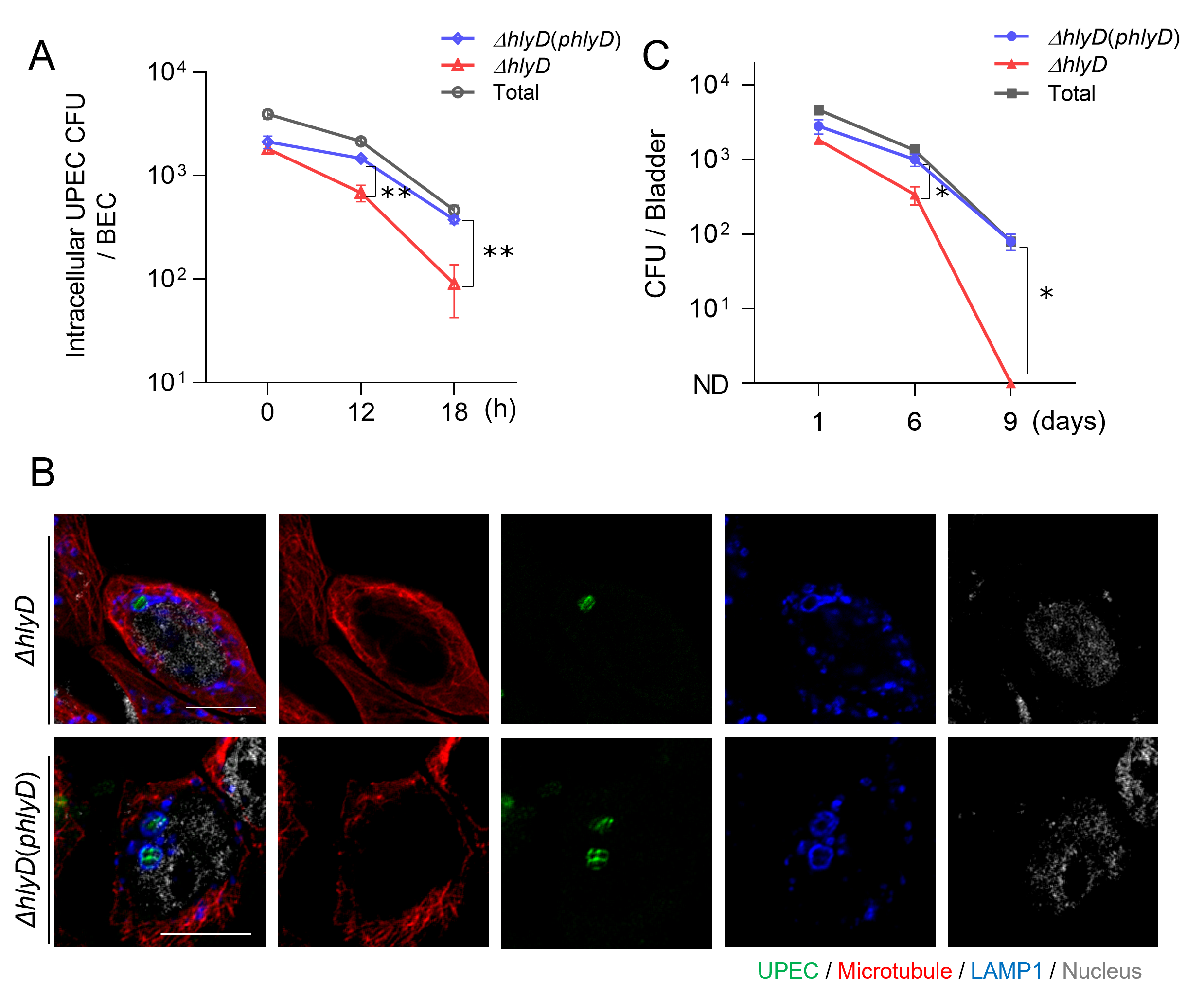

Supplement: S21 Fig — ΔhlyD UPEC strain was transformed with phlyD plasmid to generate ΔhlyD(phlyD) strain. (A) Human 5637 BECs were co-infected with equal numbers of the ΔhlyD and ΔhlyD(phlyD) strains, and intracellular UPEC numbers were examined at indicated time points. (B) After infection with these UPEC strains on human BECs, the cells were stained with anti-tubulin (red), anti-UPEC (green), and anti-LAMP1 (blue) antibodies for confocal microscopic imaging. (C) C57BL/6J was co-infected with equal numbers of the ΔhlyD and ΔhlyD(phlyD) strains and bacterial burden in mouse bladders were examined. Data were analyzed by two-way ANOVA (A, C). *P<0.05; **P<0.01, Scale bar: 10 μm. (TIF) [file ppat.1011388.s021.tif]

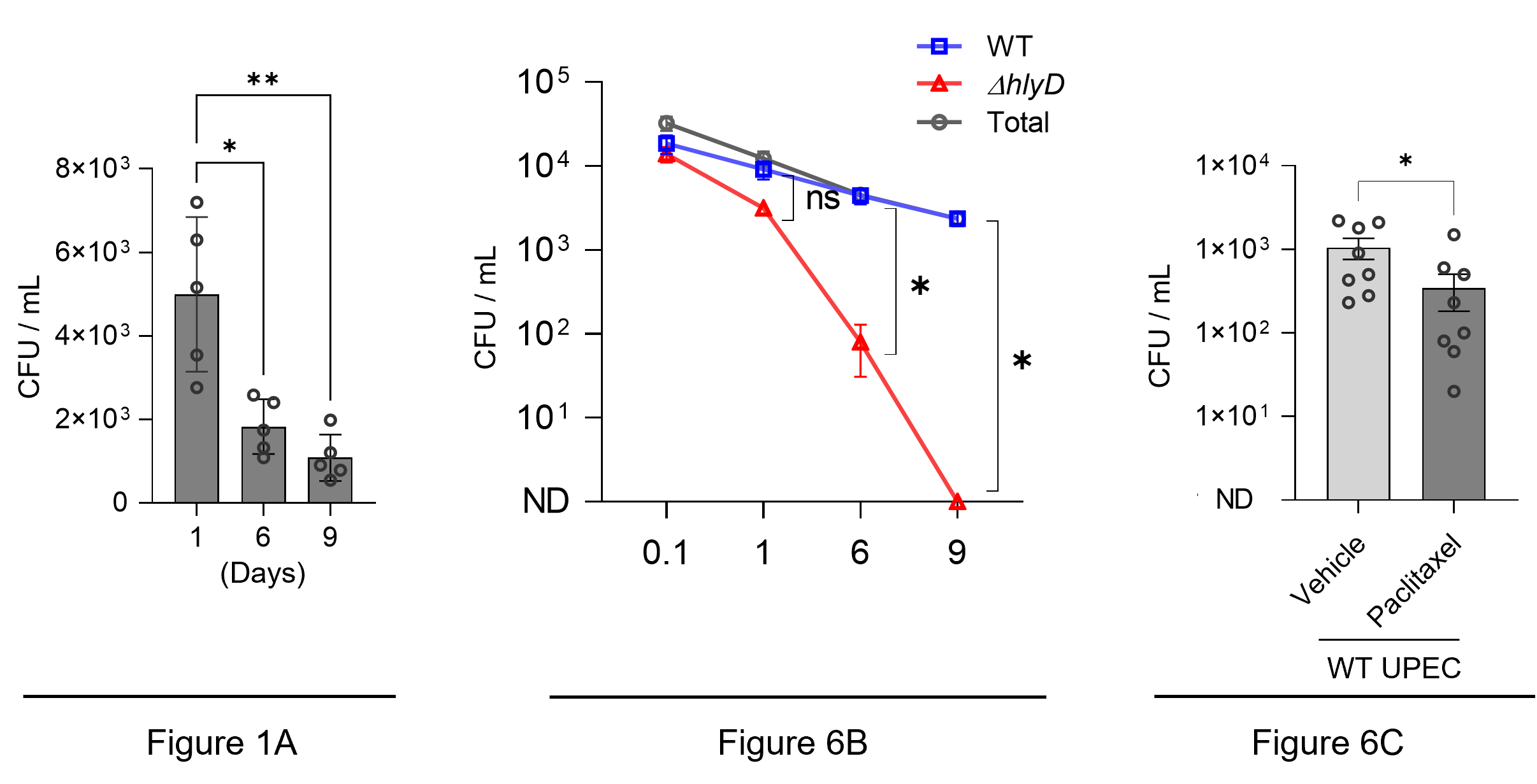

Supplement: S22 Fig — The detection limit of CFU in mouse bladders was 100 CFU per bladder. (TIF) [file ppat.1011388.s022.tif]

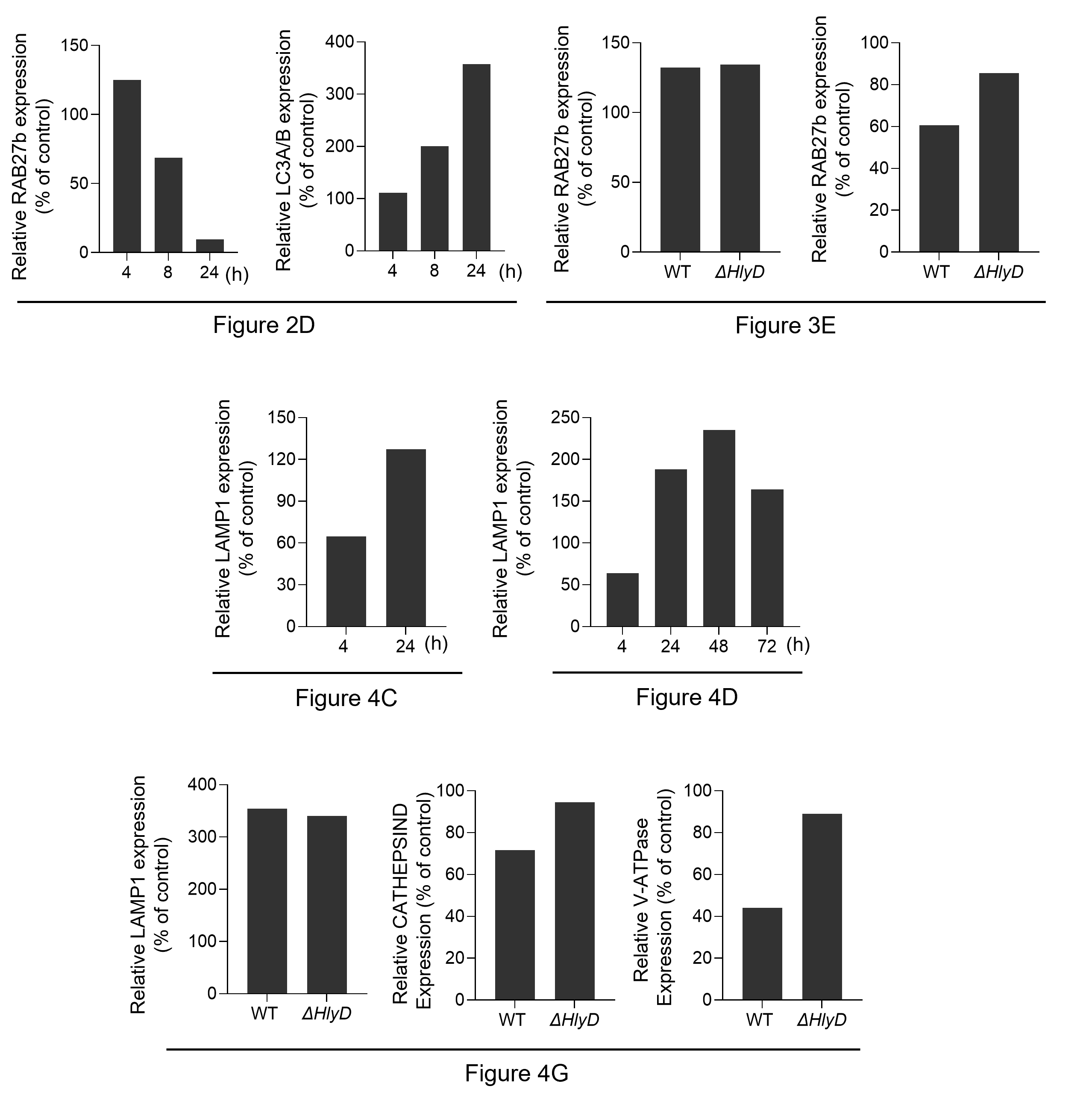

Supplement: S23 Fig — Representative results from two to three independent western blots were selected and presented. (TIF) [file ppat.1011388.s023.tif]
