# Supplementary material for: α-Hemolysin promotes uropathogenic E. coli persistence in bladder epithelial cells via abrogating bacteria-harboring lysosome acidification
Source: PLoS Pathog. 2023 May 11;19(5):e1011388. doi: 10.1371/journal.ppat.1011388 (PMC10204954; doi:10.1371/journal.ppat.1011388)
Supplement: Attachment — Submitted filename: Response to reviewers_re.docx [file ppat.1011388.s024.docx]

**Reviewer's Responses to Questions**

**Part I - Summary**

**Reviewer #1:**

**This manuscript from the Abraham lab describes life cycle of UPEC inside bladder epithelial cell in the context of UPEC persistence. More specifically, UPEC escape Rab27 b+ vesicles to become cytoplasmic using hemolysin activity; cytoplasmic UPEC are repactured by LC3A/B+ autophagosomes; and HlyA activity prevents acidification of autophagosomes into lysosomes. The authors show that hlyAKO mutant fails to leave Rab27b+ vesicles and is expelled from the cell. These results suggest the role for hemolysin activity in persistence of UPEC inside BECs.**

**Reviewer #2**:

**In this work the authors probe the intracellular dynamics of UPEC within 5637 bladder epithelial cells and the role of the UPEC HylA toxin in preventing autolysosome acidification. The authors further postulate that HylA functions to reduce V-ATPase recruitment to the autolysosome by destabilizing microtubules. The authors go on to test the effects of intravesical instillation of a microtubule stabilizer to reduce bladder bacterial burden. While this work is interesting, I do have some concerns.**

**1.** **The introduction and discussion need extensive revision for clarity, grammar, and inclusiveness of the existing literature. There are several existing papers about UPEC hemolysin A that, for example, have not been referenced or adequately discussed.**

**These include two papers https://doi.org/10.1073/pnas.150037411 (PMID: 25675528) and https://doi.org/10.1128/IAI.00075-08 (PMID: 18443089) that report that loss of HylA does not affect bladder colonization in a mouse model. The first reference specifically shows that loss of HylA did not affect intracellular populations of UPEC or IBC formation in a mouse UTI model. Also, although doi: 10.1016/j.chom.2011.12.003 (PMID: 22264513) is referenced by single sentence in the discussion, since this work was the first to report microtubule disruption by UPEC HylA (via paxillin degradation) a more thorough discussion is warranted. The authors are encouraged to more thoroughly contextualize their findings within the existing literature.**

🡺 We appreciate the reviewer's insightful comments on our manuscript from the HaeWoong Choi Lab.

Based on their suggestion, we have revised the manuscript extensively and incorporated additional HlyA research literature. Please see **Lines 69**, **369**, and **377**.

**2. I would encourage the authors to report absolute numbers of bacteria instead of just as percentages. These data can be included in the supplement. With only the percentage of UPEC in each compartment reported, for example it is impossible to know if the absolute numbers of bacteria in different compartments actually changes as the infection progresses (for example, the same number of bacteria could be in Rab27 vesicles at later time points but appear less as percentage if there are more total intracellular UPEC at later time points). In general, all data should be reported as absolute numbers so changes in populations can be more accurately assessed by the reader.**

🡺 As advised by the reviewer, we have included the absolute number of bacteria for each experiment in **supplementary Figure S8, S10, S11, S12, S13, S14, S17,** and **S18**. The absolute numbers in the various compartments do change as the infection progresses.

**Reviewer #3:**

**This manuscript builds on prior work investigating UPEC persistence within bladders. Persistent/chronic UTI has been investigated by several groups, including a possible role for HlyA hemolysin. However, this is the first to state a role for the HlyA hemolysin in lysosome acidification. The premise is interesting, but some substantial concerns need to be addressed.**

**A major strength is the variety of assays that were conducted, including tracking of magnetically labeled bacteria in cultured cells and treatments with reagents to reduce bacterial burden in both cultured cells and a mouse bladder model, although this is tempered by the uneven application of these assays across different experimental conditions. There are major concerns about the lack of description for several methods, lack of some important controls, and overstated conclusions.**

🡺 In accordance with the reviewer’s comments, we have extensively revised the manuscript by incorporating more detailed descriptions of the methods, running control experiments, and rewording the conclusions.

**Part II – Major Issues: Key Experiments Required for Acceptances**

**Please use this section to detail the key new experiments or modifications of existing experiments that should be absolutely required to validate study conclusions.**

**Reviewer #1:**

**1. Page 4 last paragraph: As many as 37.6% of UPEC isolated from first-time UTI patients are reported to express the hlyA gene [20] but the expression rate significantly increased to 48.2% in patients experiencing recurring UTIs, implying a role of HlyA in the recurrence of UTIs [20]. Provide sample size for the cohorts with first-time UTI and recurrent UTI. Discuss why UPEC isolated from only 31% of subjects with second UTI were hlyA+.**

🡺 In a pervious the study [1], all isolates from women aged 19–38 years and the number of women with first-time UTI was 285. The number of women aged 18–39 years with recurring UTI (more than three infections in the past year) was 29.

Unfortunately, the issue of a second UTI was not discussed in this paper [1]. However, we speculate that other factors such as host immune responses or antibiotic treatment may influence the difference between second and recurrent UTIs.

This information is included in the manuscript (**Line 412**)

**2. Please add scale bars to all microscopic images.**

🡺 We have incorporated scale bars in all micrographs.

**3. Circles, squares, and rectangles are used to indicate different treatments throughout the manuscript. This is confusing and must be made consistent.**

🡺 We have modified each graph so that circles (UPEC CI5 strain), squares (UPEC CFT075 WT strain), triangles (UPEC CFT073 *ΔhlyD* strain), and others (described in the manuscript) denote the same condition throughout the manuscript.

**4. Figure legends must be rewritten to include relevant information**

🡺 We have rewritten the figure legends and included relevant information.

**5. Fig 1A and C. The legend states that the data are from two-three biological replicates. How many mice were examined in each of the biological replicates? The CFU data (Fig 1A) should be presented with median as central tendency and analyzed for statistical significance using Mann Whitney U test. Also, SEM is not the correct measure of uncertainty.**

🡺 In **Figures 1A** and **1C**, each dot represents a single mouse. The data in **Figure 1A** represent three independent experiments, whereas the data in **Figure 1C** represent two independent experiments. These figures display the representative data.

As requested, we have modified the bar graph in **Figure 1A** with the median as the central tendency and SD. However, we respectfully disagree about using the Mann-Whitney U test for **Figure 1A**.

The Mann-Whitney U test is used to compare differences between two independent groups when the dependent variable is either ordinal or continuous, but not normally distributed. However, the samples shown in **Figure 1A** belong to three distinct groups. Therefore, we believe that a one-way analysis of variance (ANOVA) is a more appropriate statistical analysis for determining whether there are statistically significant differences between the means of three or more independent (unrelated) groups.

**6. Fig 1B. >50% UPEC (red) appear to be NOT associated with LAMP1+ vesicles (blue) at 9-day pi. It will be informative if the authors quantify these data are present them.**

🡺 We apologize for using a low magnification image in **Figure 1B**. We have included an image with high magnification (**Fig R1)** to illustrate the colocalization of UPEC and LAMP1 vesicles more clearly in **Figure 1B**. This figure shows that UPEC (red) and LAMP1 (blue) vesicles are co-localized in the superficial epithelial cells of the mouse bladder at the 9-day time point. To quantify UPEC and LAMP1 vesicle colocalization, we evaluated multiple regions in tissue sections (80 μm × 80 μm microscopic area) and counted the number of colocalizations. Based on this, the colocalization score was found to exceed 90% (**Figure R1;** right bottom panel). We have inserted this new data in the manuscript (**Fig 1B, bottom panels**).

Figure R1. Colocalization of UPEC and LAMP1 compartments in UPEC-infected bladder at 9 days post-infection. Bottom-right panel: Quantification of co-localization between UPEC and LAMP1^+^ vesicles in an 80 μm x 80 μm microscopic region of bladder tissue. Scale bar: 10 μm.

**7. The authors mention on page 5, “However, it is noteworthy that by day 9, no neutrophils were detected in the urine of the infected mice.” How did the authors reach this conclusion?**

🡺 This conclusion was based on the fact that the MPO levels on day 9 post-infection were comparable to those in the uninfected controls. We conducted an additional and independent experiment to support this conclusion in the revised manuscript.

Figure R2. Neutrophil infiltration in infected mouse bladder peaked at 6 h p.i. and then diminished. Recruitment of neutrophils was measured by flow cytometric analysis of the infected mouse bladders. C57BL6/J mice were infected with UPEC CI5 strain, after which harvested bladders at each time point were analyzed via flow cytometry. (A) Ly6G^+^ CD11b^+^ CD45^+^ cells in the infected mouse bladders were counted by flow cytometry. The population percentage was determined using CD45^+^ cells. N=5-8. (B) A Gating strategy to define the neutrophil population in this analysis. Data were analyzed by ordinary one-way ANOVA, ***P<0.001; ****P<0.0001.

C57BL/6J WT mice were infected with the UPEC CI5 strain and the neutrophil levels of the bladders were analyzed using flow cytometry at different time points. Neutrophils in a single-cell preparation of bladder tissue were quantified using previously described methods [2], where anti-Ly6G, anti-CD11b, and anti-CD45 antibodies were used as probes (**Fig R2**). We observed a sharp and significant elevation in the neutrophil population (Ly6G^+^ CD11b^+^ CD45^+^) at 0.25 day (6 h) post infection, which decreased by 1 day post infection. By days 6 and 9, no significant difference in neutrophil numbers was observed between the infected and uninfected mouse bladders (**Fig 1C**).

The above data have been added to the Results section. This information is included in the manuscript (**Line 98**) and in the main figure (**Fig 1C and Fig S2**).

**8. Fig 1C. Define the positive control for MPO assay. Also, present raw MPO data either as ΔOD/min/g of tissue OR by extrapolating it to a known MPO standard. The current presentation shows % relative MPO activity with 700% MPO activity at 6 hpi. This does not make sense. How many biological replicates do these data represent? Why is the sample size at 6 hpi so low?**

Figure R3. Neutrophil-specific enzyme activity peaked at 6 h p.i. and then diminished. C57BL/6J mice were infected with UPEC CI5 strain, and harvested bladders at each time point were analyzed using myeloperoxidase assay. MPO content in mouse bladder was estimated as mU per mouse bladder. N=3-13. Data were analyzed by an ordinary one-way ANOVA, ****P<0.0001.

🡺 Recombinant MPO was used as the standard to determine MPO activity and was therefore used as the positive control in this experiment. To evaluate bladder MPO levels, MPO activity was measured at OD 450 nm, and MPO content was estimated as mU per bladder by comparing the slope of each readout to that of a recombinant MPO standard. Each reading was based in four replicates. We have represented our data considering the reviewer’s comments (**Fig R3**).

As the MPO assay only measures neutrophil-specific enzymes, we performed an additional assay (flow cytometry) to measure neutrophil numbers. Please see our response to **reviewer question #7** and **Figure R2** (**Fig 1C** and **S2** in the revised manuscript **Line 98**)**.**

**9. Figure 2 legend states that the data are from two-three biological replicates. How many technical replicates in each biological replicate? Also, SEM is not the correct measure of uncertainty. Replace it with SD. Please do this for all relevant results presented in Fig 2 (panels B, C, G, H) and other figures.**

🡺 In this study, the individual dots on each graph represent biological replicates (**Fig 2E**, **2G** and **Fig 2H**). In **Figures 2B** and **2C**, the data evaluated are from three biological replicates, and individual dots represent UPEC counts in a randomly chosen optical field. Each biological replicate corresponds to a single technical replicate. In accordance with the reviewer’s suggestion, we have replaced the SEM values with SD values throughout the manuscript.

**10. Provide details for confocal microscopy quantification data in Fig 2B, C, how many total infected cells did you count to reach the % of UPEC associated with Rab27b+ vesicles? Is it correct to say that in the presented image (Fig 2A) only one out of three cells are infected?**

🡺 To quantify **Figures 2B** and **2C**, we randomly selected optical fields (100 μm × 100 μm) for confocal microscopy. Typically, we examined 4–8 optical fields at each time point, which typically contained 25–100 cells. Three biological replicates were used for each time point. Although several intracellular UPEC were observed to be either completely or partially devoid of an encasing vesicle, only UPEC that were completely encased in specific vesicles were counted in this assay. At 4 h p.i., approximately 60% cells were found to harbor intracellular UPEC when we quantified Rab27b^+^ UPEC in the 100 μm × 100 μm optical field of confocal microscopy images.

The images in **Figure 2A** in the manuscript were selected and their optical fields were magnified to clearly illustrate the morphology of UPEC and vesicles. Concerning the question posed by the reviewer, we do not believe that only one out of the three BECs was infected with UPEC. The microscopy images in **Figure 2A** were captured using a confocal microscope, which scanned only one plane of the object. Thus, there are more UPEC associated with each BEC at different focal planes. In this particular image, the optimal focal plane showing UPEC in the vesicles was selected and captured.

Figure R4. Increased intracellular UPEC loads after autophagy component knock-down. Human 5637 BECs were transfected with BECLIN siRNA, ATG5 siRNA, or NS siRNA. Transfected cells were infected with UPEC CI5 strains at the indicated time-points, and the intracellular bacterial burdens were compared. Data are shown as mean ± SD. Data were analyzed by an ordinary one-way ANOVA. *P<0.05

**11. Fig 2H shows that siRNA treatment results in 200% of infected cells to contain UPEC. What does this mean? Also, are these UPEC associated with a specific vesicle?**

🡺 The 200% refers to the increase in intracellular bacterial load in transfected BEC compared with WT BECs. Importantly, the Y-axis in the figure was computed based on CFU percentage for each condition relative to the CFU of WT BECs infected with UPEC. When BECLIN or ATG5 was knocked down in BECs, the intracellular bacterial burden increased by 200% compared with the CFU of WT BECs. For clarity, the experimental results are presented as CFU-based bar graphs in **Figure S11** (**Fig R4**).

**12. The authors should explicitly state that Fig 2 shows in vitro experiments using 5637 cell line. There is a drop in UPEC associated with Rab27b+ vesicles from 70% at 5h to 30% at 12h as shown in 2B. However, this is not supported by the data shown in Fig 2E. Please discuss this. Also, why only one early timepoint for Fig 2D and why not have similar time points for 2D and 2E?**

🡺 **Figure 2E** has been changed to **Figure** **2D** in the revised manuscript.

Figure R5. UPEC transition from Rab27b+ vesicle to LC3A/B autophagosome. Human 5637 BECs were treated with magnetic bead-labeled UPEC. After 4, 8, and 24 h of incubation, isolated UPECs were analyzed for the expression of Rab27b and LC3A/B.

As requested by the reviewer, we have indicated the use of the human 5637 cell line in the figure legend.

We agree with the reviewer regarding matching the time points in **Figures 2B** and **2D**. Therefore, we performed a western blot analysis of UPEC-infected BECs at various time points (4, 8, and 24h) (**Fig R5**). This result shows that as time progressed, the number of UPEC in Rab27b^+^ vesicles containing UPEC at the early stage of infection decreased. Additionally, western blotting with the LC3A/B antibody revealed that the number of UPEC within LC3A/B vesicles increased over time. Overall, the western blot results (**Fig 2D**) with the anti-Rab27b antibody directly correlated with the decreased expression of the Rab27b^+^ vesicles (**Fig 2B**).

The above data have been added to the Results section. This information has been included in the manuscript (**Line 141**) and in the main figure (**Fig 2D**).

**13. In Fig 2G, how can you calculate %UPEC/total infected BEC for TritonX100 treated wells? Won’t Triton treatment break open ALL cells, and total infected BEC will be 0? This is a minor concern that will be resolved by clearly explaining how the calculations are done in this and other figures such as Fig 3H.**

Figure R6. Intracellular vesicle isolation using magnetically labeled bacteria. Human 5637 BECs were seeded and infected with magnetically labeled UPECs. After lysing cells by passing the cells through needles, UPEC-containing vesicles were isolated with magnet. Isolated vesicles containing UPECs were treated with 0.1% Triton X-100 or saline, then incubated with gentamicin before plating on McConkey agar plates.

🡺 To explain the experimental procedures more clearly, we illustrated the experimental procedures (**Fig R6**) and incorporated this in the **Figure S7**. As shown in **Figure R6**, 0.1% Triton X-100 treatment of isolated vesicles only perturbs the host membrane and does not alter UPEC cell viability. To clarify this point, the Y-axis of **Figure 2G** was changed to “Bacterial survival after gentamicin / Isolated magnetic bead-UPEC (%)”. To compute the percent UPEC survival, [CFU of gentamicin-treated isolated UPEC] was divided by [CFU of isolated UPEC without gentamicin]. The experimental procedure for magnetic bead labeling of UPEC and gentamicin treatment of magnetic beads was adopted and modified from a previously published study [3].

**14. Include complemented hlyA KO CFT073 containing plasmid-borne hlyD to confirm that hlyD KO does not have polar effects. The complemented strain should be included in BEC and in vivo experiments.**

🡺 We conducted a complementation study, as suggested by the reviewer. We complemented the Δ*hlyD* UPEC strain with p*hlyD* and compared its ability to alter the intracellular survival of UPEC in human 5637 BECs and mouse bladders. We infected human BECs with equal numbers of Δ*hlyD* and Δ*hlyD*(p*hlyD*) strains and then compared their relative capacity to survive within the same BECs. We differentiated the bacterial strains during culture based on the antibiotic markers they carried. At 18 h p.i., the Δ*hlyD*(p*hlyD*) strain demonstrated a 10-fold increase in total intracellular bacteria compared to the Δ*hlyD* strain (**Fig R7A**). When these samples were imaged using a confocal microscope, the Δ*hlyD*(p*hlyD*) strain exhibited rearranged and less organized microtubules in infected BECs than the Δ*hlyD* strain (**Fig R7B**). When mice were co-infected and the survival of these UPEC was evaluated, 1 × 10^2^ CFU of the Δ*hlyD*(p*hlyD*) strain was still detectable in the bladders on day 9 p.i. whereas the Δ*hlyD* strain was not (**Fig R7C**). Therefore, the observed phenotype was not a consequence of polar effects.

Figure R7. Recovery of phenotype after complementation. Δ*hlyD* UPEC strain was transformed with p*hlyD* plasmid to generate Δ*hlyD*(p*hlyD*) strain. (A) Human 5637 BECs were co-infected with equal numbers of the Δ*hlyD* and Δ*hlyD*(p*hlyD*) strains, and intracellular UPEC numbers were examined at indicated time points. (B) After equal co-infection with these UPEC strains on human BECs, the cells were stained with anti-tubulin (red), anti-UPEC (green), and anti-LAMP1 (blue) antibodies for confocal microscopic imaging. (C) C57BL/6J was co-infected with equal numbers of the Δ*hlyD* and Δ*hlyD*(p*hlyD*) strains and bacterial burden in mouse bladders were examined. Data were analyzed by two-way ANOVA (A, C). *P<0.05; **P<0.01, Scale bar: 10 μm.

The above-mentioned data have been added in the result section. This information is included in the manuscript (**Line 331**) and has been added in the figure (**Fig S19**).

**15. Label x-axis in Fig 3C. Please define it in the figure legend as well. Not clear how you reached MOI of 50 from Fig 3C.**

🡺 The missing X-axis label has been incorporated in **Figure 3C**. Additionally, we have specified that the X-axis represents the MOI.

Regarding the selection of MOI 50, we selected this particular MOI based on the cytotoxicity assay result shown in **Figure 3C**. Compared to the negative control, MOI 50 was selected as it falls between an MOI where statistically significant cytotoxicity was observed and an MOI with no statistically significant cytotoxicity. After selecting this MOI, we experimentally confirmed that MOI50 lacked cytotoxicity (data not shown).

Figure R8. Bacterial burden comparison on C57BL/6J vs C3H/HeN mice. After 24 h of infection with UPEC CI5 strain, the mouse bladder and kidney from C57BL/6J or C3H/HeN were harvested and examined for CFUs. Data were analyzed by unpaired two-tailed Student’s t-test. ****P<0.0001; n.s, not significant.

**16. Show limit of detection with a dotted line in Fig 6.**

🡺 As shown in **Figure 6B**, the homogenized bladders were diluted by a factor of 50 and subsequently plated on a MacConkey plate. In all samples (n=5 mice bladders), the Δ*hlyD* strain infection rate was zero.

**17. Did you detect any CFUs in kidneys?**

🡺 In our mouse model of UPEC-mediated cystitis, no UPEC colonization was observed in mouse kidney because we instilled UPEC in a small volume with minimal pressure (2.5 μL/sec) to avoid a spontaneous retrograde flow (vesicoureteral reflux) from the bladder to the kidney [4]. To confirm the validity of our mouse model, we conducted control experiments using C57BL/6J and C3H/HeN mice. **Figure R8** shows that the inoculum did not reflux into the kidney.

Figure R9. Human 5637 BECs were treated with magnetic bead-labeled UPEC CI5 strain or UPEC CI5 strain (unlabeled). Intracellular CFU were examined after 2 h of incubation with gentamicin to remove extracellular UPEC. ns: not significant.

**18. Fig 6C, right panel. What is the meaning of y-axis label here?**

🡺 We have clarified the label on the Y-axis. We have replaced “(%) CFU of total UPEC / Bladder” with “Relative to vehicle treatment (%)” on the right axis.

**Reviewer #2:**

**1. While the use of magnetic bead labeled UPEC is a new an interesting approach, there are experiments lacking which are necessary to validate this system. I don’t see data in this manuscript demonstrating that labeling UPEC with magnetic beads does not alter how UPEC attaches to and invades BECs. Are recovered CFUs similar to what is observed with unlabeled UPEC? Do the magnetic bead UPEC express type 1 fimbriae and other virulence factors (like HylA) similarly to unlabeled UPEC? Also, for all of the magnetic pull down experiments, a “vehicle” control where magnetic beads are added to BECs (without UPEC) needs to be performed. This is important as a non-specific binding control to determine if the beads are taken up into vesicles by themselves (independent of UPEC) and if they alone can pulldown RAB27b fusiform vesicles for example. Also, when performing these types of experiments, it is usually good practice to perform blots for markers of other cellular compartments you do not expect the bacteria to be housed in to ensure specificity.**

Figure R10. Human 5637 BECs were treated with magnetic bead-labeled UPEC CFT073 strain or UPEC CFT073 strain (unlabeled ). After 16 h of incubation, percent release of lactate dehydrogenase was analyzed. ns: not significant

🡺 In this study, we used a previously validated and published assay system [3]. In this assay, *E. coli* was cross-linked to carboxyl magnetic particles, enabling convenient isolation of intracellular *E. coli* after host cell membrane lysis.

To address some of the reviewer’ concerns, we have now included additional assays. First, we exposed human 5637 BECs to comparable numbers of unlabeled type fimbriated UPEC and magnetic bead-labeled type 1 fimbriated UPEC in tissue culture wells. After 2 h of incubation with gentamycin to kill extracellular bacteria, we assayed different incubation wells for intracellular bacteria and found no significant difference in the numbers of labeled and unlabeled bacteria (**Fig R9)**, which confirmed that magnetic particle labeling did not affect the ability of UPEC to bind and invade BEC. Second, we repeated the previous experiment, except that this time, we allowed the incubation to proceed for 16 h, which enabled intracellular bacteria to produce cytotoxic HlyA. **Figure R10** shows the percent cytotoxicity of BECs incubated with unlabeled and labeled UPEC. We observed no significant difference in the percent cytotoxicity assessed by LDH release (**Fig R10**), suggesting that the production of hemolysin was comparable in both labeled and unlabeled UPEC.

Figure R11. Isolated magnetic beads-labeled UPEC still express FimH and are associated with Rab27^+^. Human 5637 BECs were incubated with magnetic beads alone or with magnetic beads-labeled UPEC. After 2 h of incubation, isolated beads or cell lysates from each condition were analyzed for the expression of RAB27b, Endobrevin/VAMP8, and FimH. Gapdh was used for loading control of UPEC-containing vesicle.

Third, we specifically isolated intracellular UPEC and demonstrated that they are associated with Rab27b^+^ vesicles and FimH (a component of UPEC type I fimbriae). In this experiment, human BEC were either incubated with magnetic beads alone or with magnetic bead-labeled UPEC. After 1 h of exposure, we washed BECs with gentamicin-containing media to remove extracellular UPECs and incubated the in fresh gentamicin-containing media. After two more hours of incubation, BECs in both incubation groups were lysed and a magnet was used to pull down the magnetic beads or bead-labeled UPEC for western blotting. **Figure R11** shows that although magnetic bead-labeled UPEC still expressed type 1 fimbrial protein, FimH, and was associated with the membrane protein Rab27b, naked magnetic particles were not associated with any of these markers as they presumably were not internalized by BECs.

Additionally, we demonstrated the specificity of the pull-down assay by probing for endobrevin/VAMP8 a protein primarily associated with perinuclear vesicles and typically found in early endocytic compartments [5]. Rab27b and endobrevin, but not FimH, were equally detected in BEC whole cell lysates in UPEC infected BECs. The absence of endobrevin in magnetic bead-labeled UPEC western blotting confirmed the specificity of the pull-down assay. To further confirm the specificity of these pull-down assays, whole-cell lysates from each group were subjected to pull-down assays using the same antibody probes on western blots. As expected, Rab27b and endobrevin, but not FimH, were detected in the BECs exposed to magnetic beads alone.

This information has been included in the manuscript (**Line 133**) and in the figures (**Fig S5** and **S6**).

**2. Maybe I am missing something, but I don’t understand why it is assumed there is a cytosolic intermediate for UPEC in BECs. The authors interpret the data in figure 2 to mean that UPEC escapes from RAB27b vesicles into the cytosol where it is recaptured into LC3A/B compartments. Couldn’t an alternate hypothesis be that the UPEC-containing RAB27b vesicles are trafficked and develop into LC3A/B compartments as part of the endocytic pathway? Even with evidence for a cytosolic stage (which I believe is in Figure 2G but it’s hard to tell because this figure is not called out where this is discussed in the results), how could one be certain that the bacteria individually transition from RAB27b to cytosol to LC3A/B compartments without a time-lapse experiment that tracks individual bacteria over time?**

🡺 As the reviewer’s question pertains to one of the major points in this manuscript, we are compelled to provide more convincing experimental evidence to support our claim that UPEC encased in Rab27b transition into autophagosomes, and that this transition is facilitated by the escape of UPEC from Rab27 compartments into the cytosol, albeit for a limited period.

First, as suggested by the reviewer, we incorporated more time points to track the intracellular journey of UPEC after uptake within Rab27b^+^ vesicles. As described earlier, the UPEC CI5 strain was conjugated with magnetic beads and exposed to human BECs, followed by the isolation of UPEC and associated vesicular compartments at various time points (4, 8, and 24 h). When we probed the isolated intracellular UPEC with anti-Rab27b and anti-LC3A/B antibodies (an autophagosomal marker) on western blots, UPECs were observed to be housed mostly in Rab27b^+^ vesicles at 4 h, with reduced amounts of UPEC detected in LC3-containing vesicles (**Fig R5**). However, the number of UPEC in Rab27b^+^ vesicles decreased drastically by 24 h, while the number of UPEC in LC3A/B^+^ vesicles correspondingly increased (**Fig R5**).

Second, we demonstrated the existence of an “intermediary phase”, wherein UPEC was detectable in the cytosol and devoid of an encasing vesicle. Based on the data shown in **Figure 2B**, we suspect that this occurs between 5 and 12 h post infection of BECs (**Fig 2B**). To demonstrate the presence of UPEC in BEC cytoplasm, we conducted a gentamicin protection assay on intracellular UPEC isolated via their association with magnetic beads. We reasoned that if bacteria were encased in either Rab27^+^ or LC3A/B^+^ vesicles, they would be protected from gentamicin, whereas if they were membrane-free in the cytosol, they would be susceptible to the antibiotic. As shown in **Figure R12,** we observed that 2 h post infection, when UPEC were presumably encased in Rab27 compartments, 67% of UPEC appeared to be protected. Similarly, at 8 h post infection when several of the UPEC were presumably encased in LC3A/B compartments, we observed that 77% of UPEC were insensitive to gentamicin. Interestingly, at 4 h post infection we found that only 48% of the intracellular UPEC were insensitive to gentamicin, which is consistent with the notion that at this time point, most bacteria were free in the cytosol and no longer protected from the antibiotic.

Figure R12. Time course survival of isolated UPEC from gentamicin treatment. Human 5637 BECs were infected with magnetic beads-labeled UPEC. After various time points, magnetic bead labeled UPEC were isolated from infected host cells and exposed to gentamicin (open square). No gentamicin treatment on isolated UPEC was used to calculate intracellular UPEC inside or outside of vesicles. Data were analyzed by an ordinary one-way or two-way ANOVA. *P<0.05, **P<0.01.

We believe that these time-lapse experiments employing a gentamicin protection assay support the hypothesis that there exists a transitional period between the Rab27^+^ and the LC3A/B^+^ vesicle phase, where intracellular UPEC are found free in the cytoplasm.

This information is included in the manuscript (**Line 148**) and in the main figures (**Fig 2E** and **S8**).

**3. For lanes to be qualitatively compared in a Western blot they must be on the same blot (upper and lower panels of 3E cannot be compared with each other for example as the exposure time or amount loaded seems to be different). These blots need to be redone in this configuration if comparisons are to be made. Also, the authors overstate the observed differences in band intensities in figures 3E and 4G.**

Figure R13. Reduced association of UPEC in RAB27b^+^ vesicles. The human 5637 BECs were infected with magnetic beads labeled with WT or *ΔhlyD* CFT073 UPEC strain. At 5 h p.i., the UPEC-housing vesicles were extracted and immunoblotted using an anti-RAB27b antibody. Loading control of UPEC-containing vesicles was detected with anti-GAPDH antibody.

🡺 As suggested by the reviewer, we repeated the experiments to demonstrate consistency of the result with **Figure 3E** (**Fig R13**). Additionally, we toned down the results to be objective.

**4. I am concerned about the observed CFU in the mouse bladder infection experiments in Figures 1 and 6. These are too low for and do not reflect is normally observed with UPEC infection of C57BL6 mice, especially for CFT073 (Figure 6). Given these abnormally low bladder bacteria titers, it is difficult to interpret the results of the mouse experiments. Perhaps the bladders are not being properly inoculated or there is a difference in the how the bacteria are being prepared for inoculation?**

🡺 We respectfully disagree with the notion that our CFU results represent abnormally low titers of bladder bacteria due to improper inoculation during the experiment. This assertion is based on the following points:

(i) We reviewed previously published studies describing the bacterial load in C57BL/6J mice infected with UPEC CFT073. Although some studies have revealed a higher bacterial burden than ours, other studies have reported similar findings at 24 h or 7 days post infection in C57BL/6J mice infected with the UPEC CFT073 strain [6, 7]. Thus, our results are not abnormal compared to those in the published literature [3].

(ii) We conducted an in vivo experiment to compare the bacterial burden in WT C57BL/6J and C3H/HeN mice following infection with the UPEC CI5 strain (**Fig R8**). The C3H/HeN mouse strain is often used to study acute bacterial cystitis and has been reported to have high bacterial bladder burdens relative to other mouse strains due to genetic variation in their innate immune responses (TNF-α and IL-1β levels) [8, 9] and because they experience reflux [10]. We observed a tenfold increase in bacterial burden in C3H/HeN mice with the UPEC CI5 strain compared to C57BL/6J mice (**Fig R8**), which is consistent with the results of previous studies and suggests that there is nothing wrong in our inoculation technique and that our findings are reproducible and clearly not abnormal.

**5. A critical control is missing for Figure 1D. Without the inclusion of a non-specific control (secondary antibody only), it is impossible to determine if the observed fluorescence is bacteria or autofluorescence which is common in bladder tissue. By just looking at the size, it seems as if these structures are quite a bit larger than the bacteria observed in the BECs in Figure 2A for example. Also, unless this was the only case and only field in which signal was detected the authors should provide additional representative images from other fields and other patient biopsies in the supplement to further support these findings.**

🡺 We have inserted scale bars into our images in accordance with the reviewer’s comments. The size of the *E. coli* clusters was less than 1 μm, which are consistent with the size of *E. coli*. We also included a control for this figure. Additionally, we have now included bladder tissue from patients with no history of UTI in the past two years stained with anti-*E. coli* antibody. Although no non-specific labeling was observed (**Figure 1D**), strong labeling of E-cadherin on the urothelial cell surface was observed (**Figure R14**).

Figure R14. Bladder biopsies were obtained from patients with recurrent UTI or patients with no history of UTI in the last two years. These tissues were immunostained for UPEC (*E. coli*, green), urothelium (E-cadherin, blue), lysosomal vesicle (LAMP1, red), and nucleus (DAPI, gray). “L”: lumen of the bladder. Scale bars: 10 μm.

Moreover, we have included images of more human bladders obtained from patients with UTIs. As shown in **Figure R14**, there is little evidence of non-specific anti-*E. coli* antibody staining.

This information is included in the manuscript (**Line 107**) and in the figure (**Fig S3**).

**Reviewer #3:**

**1. Different methods were applied to very narrow time points, and the findings would be more convincing if all salient markers (Rab27b, LC3A/B) were measured at each timepoint to show how they are changing together—you could use a ratio of each marker normalized to a control. How many times were these pulldowns/blots conducted? Densitometry from several blots can be shown alongside a single blot. If bacteria are supposed to be in the cytosol vs vesicles at specific timepoints, show gentamicin sensitivity changing over time instead of providing a single snapshot. Data should be provided for 1) total CFU, 2) intracellular CFU, and 3) cytosolic vs vesicle-associated CFU so it is clear whether bacteria are invading and surviving normally.**

🡺 To address the critical points raised this reviewer, the following were undertaken.

(i) We have included densitometric data of the western blots in the manuscript (**Fig S20**). Each western blot was repeated thrice and used to generate the densitometric data. A representative image was selected and is shown in Figures 2D, 3E, 4C, 4D, and 4G.
(ii) To address the need to synchronize the use of salient markers and demonstrate the dynamic nature of the intracellular trafficking of UPEC following internalization in BECs, we concurrently used Rab27b and LC3A/B probes and included more time points in our assays (**Figure R5**). These new data support and strengthen our earlier argument that UPEC harbored in Rab27b vesicles is released into the cytosol before it is once again taken up in LC3A/B vesicles (**Figure R12**).
(iii) Furthermore, we conducted an additional assay to confirm that cytosolic UPEC are readily taken up by LC3A/B autophagosomes. By comparing the gentamicin sensitivity of magnetic bead-labeled UPEC isolated from control-transfected BECs and from BECs where ATG5 was knocked down, we showed that UPEC in ATG5 knockdown BEC were significantly more susceptible, revealing that in the absence of the autophagosome machinery, UPEC remained in the cytosol and was highly susceptible to gentamicin exposure (**Figure 2G**).

This information has been included in the manuscript (**Line 148**) and in the main figures (**Fig 2E and S8**).

**2. Additional (or better) controls would also strengthen the conclusions. For example, uninfected or mock-infected cells are an important control that needs to be added for most of the experiments (see especially Fig 4). Throughout the manuscript, GAPDH is used as a control for western blots (as stated in the Fig 2 legend, although this antibody was not mentioned in the list of antibodies in the methods), but this antibody typically reacts with both mammalian and bacterial GAPDH. (note, the control should be stated clearly for the blot in Fig 3E.) Thus, it is not clear what is being assayed when, for example, magnetically labeled bacteria are used to pull down host cell components. Separate controls should be used to show both host cell protein levels and bacterial enrichment. The anti-E. coli antibody that seems to have been used for immunofluorescence microscopy might be a suitable alternative.**

🡺 We apologize for not including appropriate controls in the pull-down experiments. We believe that we have addressed all the points raised by the reviewer, including the inclusion of meaningful controls in **Figure R11.** Please see also our **response to point #1 of Reviewer 2** as well.

**3. There needs to be more work to place this study into the body of knowledge for UPEC-bladder urothelium interactions. For example, quiescent intracellular reservoirs (QIRs) have been described in chronic/recurrent UTI mouse models. Similarly, the cell line used here is derived from a carcinoma and is known to have altered autophagic response. These cells also lack the fusiform vesicle physiology of umbrella cells, and they do not assemble urothelial plaques. It is not clear if the mechanism translates to intact, polarized, uroplakin crystal-coated bladder cells. Likewise, use of pan-inhibitors will be tremendously disruptive of normal cellular physiology, including the pathways and markers in this current study. Thus, claims that specific pathways are being targeted by bacteria are overstated. This does not negate the experiments conducted, but it needs to be acknowledged.**

🡺 As suggested by the reviewer, we have now mentioned QIRs in UTI mouse models and how they could relate to our current findings, **[Introduction, Line 69]**, as well as and the limitation of using human 5637 BEC lines **[Discussion, Line 408]**. We have also cautioned that the specific pathways revealed by our studies may be affected by the cell line used.

**4. HlyA is not produced by the majority of UPECs (e.g., 31-48% PMID: 26299820, 20% PMID: 28330863) and, if anything, seems to be more correlated with pyelonephritis. Indeed, the prevalence of hlyA and its role in recurrence is overstated in the introduction: ref 19 is a lung study; ref 20 is PCR positivity, not expression, and does not demonstrate a significant increase in hlyA in patients with recurrent UTI. Thus, this proposed mechanism, if true, is restricted to a narrow subset of UPECs. It would be useful to show mouse bladder persistence for a panel of wild-type UPEC isolates as well as the 8-week persistence data that is mentioned in the first paragraph of the results but not shown. Do hlyA-negative UPEC isolates display similar persistence in the mouse model used here? This should be factored into the vaccine discussion as well; how likely is a secreted hemolysin that seems to exert its most potent action intracellularly to be an effective vaccine antigen? Finally, it is not clear how a pore-forming toxin is interfering with recruitment of V-ATPase to lysosomes. It would be helpful to propose a model that could be tested.**

Figure R15. Prolonged bacterial burden in the infected mouse bladders. C57BL/6 female mice were infected by intravesical instillation of the UPEC CI5 strain and bacterial CFUs in the infected bladders were measured at 8 weeks post-infection.

🡺 We respectfully disagree with the reviewer’s assertion that HlyA is not a prominent virulence factor in UTI isolates. Several major publications, including those by O’Hanley P, et al. in N Engl J Med [11], point to the frequency of hemolysin production among UTI isolates to be 58%, whereas studies by Orskov I, et al. and Sandberg et al. revealed incidence rates of 40% and 49%, respectively [12, 13]. It must be pointed out that the reference [14] highlighted by the reviewer where hemolysin production incidence rate was only 20% involved both symptomatic and asymptomatic UTI patients.

To address the reviewer’s request to see data on bacterial persistence in the bladder after 8 weeks of infection we present **Figure R15** (**Fig S1**), where approximately 100 CFU per bladder was cultured 8 weeks p.i. We found that the HlyA-isogenic mutant strain failed to persist for past 9 days (**Figure 6B**), indicating the key role of HlyA in promoting bacterial persistence in the bladder. We felt that incorporating persistence data for a panel of unrelated UPEC strains at this juncture was unwarranted and distracting and therefore omitted it.

Regarding the reviewer’s question as to whether a secreted hemolysin that appears to exert its most potent effects intracellularly can be an effective vaccine antigen. Notably, an alpha-hemolysin vaccine for pyelonephritis in combination with Freund's adjuvant significantly prevented renal injury in immunized mice [15]. Furthermore, T cells that can attack bacteria-infected cells can provide efficacious protection against UTIs [16].

This information is included in the manuscript (**Line 417**).

We have now included a hemolysin-mediated pathogenesis model as suggested by the reviewer (illustrated image, **Fig R16**).

Figure 16. Mechanism of UPEC persistence

**5. Please add size bars to all micrographs. In addition, bacteria are nearby the host cell vesicular membrane markers, but not colocalizing (this would mean the markers are superimposed, not adjacent). The figure legends state that confocal microscopy was used; z-stacks should therefore be collected to show that bacteria are inside vesicles. How many fields were examined for each set of experiments?**

🡺 We have now added scale bars to all micrographs. **Figure R17** shows human BECs 24h p.i. with the UPEC Δ*hlyD* strain and stained with anti-LAMP1 and anti-*E. coli* antibody. The Z-stack images of this preparation were generated using a confocal microscope. When a single plane in the z-stack was viewed along the xy-, yz-, and xz-planes it was clear that the UPEC (green) was encased in the LAMP1 vesicles (red).

Figure R17. UPEC localization in LAMP1 vesicles. Human 5637 BECs were infected with UPEC Δ*hlyD* strain. After 24 h p.i., cells were stained with anti-*E. coli* (green), anti-LAMP1 (red), and anti-tubulin (blue) antibodies. Images were captured with confocal microscope in Z-stacking mode and projected into the XY plane to visualize whole UPEC and LAMP1 vesicles (Left panel). A single plane in the z-stack was chosen and viewed along xy-, yz-, and xz-plane.

The data presented in **Figures 2A**, **2F**, **4A**, **4E**, and **4H** were generated from three independent experiments. The data in **Figure 2A** and **2F** are representative images of 6–8 fields.

**6. Please add discussion about possible toxic effects of microtubule stabilization on the bladder, particularly since this is being proposed as safer than antibiotic treatment. In addition, the current work must be better placed into the context of HlyA and bladder persistence work done by others in the UTI field (PMID 25675528). It would be helpful to discuss how Rab27b+ positivity, which is used for apical targeting of fusiform vesicles (e.g., PMID 27009205), is tied to bacterial internalization.**

🡺 We agree with the reviewer that paclitaxel is associated with side effects when administered intravenously as a chemotherapeutic agent. These side effects include pseudoallergic reactions, such as rash, hives, itching, and swelling. However, we suspect that these side effects do not occur if the drug is administered intravesically. In our mouse studies, intravesical administration of paclitaxel did not result in any adverse reactions, including hematuria, pain-associated behavior, or core-body temperature changes.

The reviewer is correct in stating that Rab27b transports fusiform vesicles toward the plasma membrane, where VAMP8/endobrevin facilitates fusion with the apical surface of urothelial umbrella cells to increase bladder volume to accommodate urine [17]. UPEC appears to hijack this Rab27b-mediated vesicle recycling pathway to gain entry into BECs and persist within BECs for extended periods [18].

This information has been included in the revised manuscript (**Discussion,** **Lines 364** and **389**).

**Part III – Minor Issues: Editorial and Data Presentation Modifications**

**Please use this section for editorial suggestions as well as relatively minor modifications of existing data that would enhance clarity.**

**Reviewer #1: 1. On page 6, please replace capacity with ability in the following sentence: “Thus, the capacity of UPEC CI5 to persist within human BECs in vitro appeared to mimic their capacity to persist within the superficial bladder epithelium in mice.”**

🡺 We have changed the word in accordance with the reviewer’s suggestion.

**2. Please rewrite the following sentence, it is very difficult to understand.**

**Page 6- “These findings suggest that evading vesicles that encase UPEC can benefit the bacteria, as they assist in avoiding elimination by BECs.”**

🡺 We have deleted this sentence and rewritten this paragraph (**Line 123**).

**3. Page 7: “Bacterial growth was minimal in siRNA-transfected BECs, but was significantly higher in BECs where autophagosomes were deficient and many more cytosolic bacteria were present (Fig 2H).” This sentence is difficult to understand. Minimal in reference to what? Which siRNA transfected BECs show minimal bacterial growth? Also “many more” is vague, please replace it with by “X-fold higher or lower”.**

🡺 We have deleted this sentence and rewritten the sentence (**Line 166**).

**4. “then at a time when we expect UPEC to be cytosolic, solubilize BECs to release intracellular BECs and then isolate them using a magnet.” Correct this sentence.**

🡺 We have deleted this sentence and rewritten this paragraph (**Line 148**).

**5. Rephrase the following sentence. It is difficult to understand: “To prove that these isolated UPEC are not encased in any vesicle and are truly cytosolic, we exposed them to gentamicin, and expected them to be susceptible to gentamicin because they are not membrane-bound.”**

🡺 We have deleted this sentence and rewritten this paragraph (**Line 148**).

**6. Page 8: “we labeled the WT CFT073 and ΔhlyD mutant strains with magnetic beads and exposed them to BECs at an MOI of 20, for 2 h.” Exposing bacteria to BECs sounds counterintuitive. Rephrase this.**

🡺 We have rewritten the sentence (**Line 191**).

**Reviewer #2: 1. Scale bars are missing from all micrographs.**

🡺 We have added the scale bars to all the micrographs.

**2. Please provide a reference for the selective disruption of autophagosome membranes by Triton X-100**

🡺 We apologize for misstating what we did. We meant to say “0.1% Triton X-100 selectively disrupted host membranes without affecting UPEC viability”. The use of 0.1% TritonX-100 for this purpose has been reported in the literature [3].

This information is included in the manuscript (**Line 164**).

**3. Line numbers should be added**

🡺 We have added line numbers.

**4. Please explain more clearly what ATG5 is and why exactly knocking it down would reduce the number of autophagosomes – saying it is an essential component is not sufficient.**

🡺 We have added more details about ATG5 and LC3A/B in the Results section (**Line 156**).

**5. For the experiment in Figure 4H, I think it would be more appropriate to pharmacologically inhibit the V-ATPase after infection has been initiated then to perform siRNA knockdown before the infection is started.**

Figure R18. Pharmacological inhibition of V-ATPase. Human 5637 BECs were infected with UPEC Δ*hlyD* strain, and extracellular UPEC was removed by gentamycin treatment. Subsequently, infected BECs were incubated in media containing Bafilomycin A1, a known pharmacological inhibitor of V-ATPase (vacuolar H+-ATPase), at 100 nM concentration. After 24h post-treatment, LysoTracker^TM^ Red DND-99 dye was applied and then stained with anti-LAMP1 (blue) or anti-UPEC (green) antibodies for confocal microscopy imaging. The number of UPEC co-localized with LysoTracker^TM^ Red dye was quantified in randomly chosen fields. Data were analyzed by unpaired two-tailed Student’s t-test. ****P<0.0001. Scale bar: 10 μm.

🡺 In accordance with the reviewer’s suggestion, a pharmacological inhibition experiment was conducted to support the gene knockdown results presented herein.

In this experiment, Bafilomycin A1, a known pharmacological inhibitor of V-ATPase (vacuolar H^+^-ATPase) was used to treat human 5637 BECs infected with the UPEC Δ*hlyD* strain. The acidification of UPEC-containing LAMP1^+^ vesicles were examined using a confocal microscope. **Figure R18** shows that V-ATPase inhibition in host BECs results in the loss of LAMP1^+^ acidification, which is consistent with **Figure 4H**.

This information is included in the manuscript (**Line 253**) and in the figure (**Fig S16**).

**Reviewer #3: 1. Fig 1B/C: bacteria that persist in the bladder could also be resistant to neutrophils, perhaps due to biofilm formation, fast growth, or toxins that directly affect neutrophils. Indeed, HlyA has been previously hypothesized to suppress inflammatory responses (PMID 22264513, which was cited in the discussion but not addressed in this context).**

🡺 These sentences were added in the Discussion section in accordance with the reviewer’s suggestion (**Line 381**).

**2. LC3A/B should appear as a doublet, but only one band is shown in the very closely cropped image (the lower band might be barely visible). The ratio between these is useful to distinguish autophagocytic response. It would be helpful to show the full blot as a supplemental figure. Also, Fig 1E describes experiments out to 5 days p.i.; is it surprising that bacterial numbers in this cell culture model remained steady after 24h? Were intracellular bacterial communities ever observed?**

🡺 We have now updated the western blot image showing LC3A/B, where a doublet is shown (**Fig 2D**)

We did not detect a significant increase in the number of UPEC after 24 h and even 7 days post infection (unpublished data). Notably, even if IBCs are formed in BECs, they are unlikely to alter the CFU because daughter cells do not separate from the parent cell.

**3. The y-axis for several figures (e.g., Fig2B and C, Fig 3F and H) is confusing. It seems that the denominator is “total UPEC” but the axes show “/Total infected BECs.” If it is indeed total UPEC, how is that being quantified? Please clarify.**

**🡺** We have elucidated the labeling of the figures. In **Figure 2B**, "total infected BECs" has been changed to "total number of infected BECs”. Although the majority of BECs in **Figure 2B** were infected with UPEC, some cells lacked UPEC. Therefore, we randomly selected and counted the number of UPEC-containing BECs by examining the confocal microscopy images.

In **Figure** **3F** and **3H**, “total infected BECs” has been changed to “total UPEC”.

**4. Figs 3A, B, C: please describe how hemolysis and cytotoxicity were assayed. Both techniques are absent from the methods section.**

🡺 We now described the methodology of both assays to the materials and methods section.

**5. Fig 4D: Please comment on the apparent increase in LAMP1 at 48h.**

🡺 We believe that autophagosomes harboring UPEC continuously recruit LAMP1 vesicles to kill UPEC until 48 h p.i. However, continuous fusion of LAMP1 vesicles will results in abnormal LAMP1 accumulation, which may necessitate LAMP1 vesicles to divide the enlarged LAMP1^+^ vesicles to maintain homeostasis. Little is known about lysosome fission, which is required to maintain the steady-state number, size, and function of lysosomes and to balance the constant flow of cargo into lysosomes [19]. We assumed that lysosomal fission decreases the size of LAMP1 lysosomal vesicles, therefore, LAMP1 expression at 72 h may decrease. Consequently, when we isolated magnetic bead-labeled UPEC at different time points, the enlarged status of LAMP1 vesicles at 48 h post infection may have differed from the divided status of LAMP1 vesicles at 72 h post-infection.

**6. Fig 4G: what is this time point? This is especially important since Fig 3 suggests there are different levels of wt vs mutant bacteria by 4 hpi.**

🡺 The time point in **Figure 4G** is 24 h post-infection.

**7. Fig 6: methods need to be provided detailing the dosages and timing of paclitaxel administration.**

🡺 C57BL6/J mice were infected with the UPEC CFT073 WT strain. After 12 h of UPEC infection, paclitaxel (5 mg/kg) or vehicle was administered intravesically. Two additional doses of paclitaxel or vehicle were administered 24 h apart. After 24 h of the last administration, the mice were sacrificed and their bladders were harvested to evaluate the bacterial load. Regarding vehicle, paclitaxel was dissolved in 50% PEG 400 solution (prepared in 0.9% saline solution).

These methods were added to the Materials and Methods section.

**8. Methods: Please provide a reference for UPEC strain CI5 or provide further description of its origin.**

**As far as I am aware, Dr. Mobley is still at the University of Michigan, not Washington. Was bacterial viability measured after magnetic labeling? What percentage of bacteria were labeled by the beads? Please note inconsistent spelling of “gentamicin” throughout.**

**🡺** We have inserted several references [20-22] in the Materials and Methods section, where the UPEC strain CI5 was used. The complete genome sequence of this strain is provided in one of these references.

We have now corrected Dr. Mobley's affiliation.

We did not directly examine the viability of the magnetically labeled UPEC. The procedure involves crosslinking of carboxyl group magnetic particles with amine residues on the bacteria, which is presumed to be relatively innocuous. Nevertheless, we indirectly examined the viability of magnetic bead-labeled UPEC by comparing the number of intracellular magnetic bead-labeled UPEC with that of intracellular unlabeled UPEC. As shown in **Figure R9**, no significant difference was observed in the intracellular bacterial burden between the two groups after 2 h of infection. Based on the data presented in **Figures R9 and R10**, we believe that most of the UPEC were labeled by magnetic beads.

We have rectified inconsistent spelling, typos and errors throughout the manuscript.

**References**

1.	Marrs CF, Zhang L, Tallman P, Manning SD, Somsel P, Raz P, et al. Variations in 10 putative uropathogen virulence genes among urinary, faecal and peri-urethral Escherichia coli. J Med Microbiol. 2002;51(2):138-42. Epub 2002/02/28. doi: 10.1099/0022-1317-51-2-138. PubMed PMID: 11863265.

2.	Choi HW, Bowen SE, Miao Y, Chan CY, Miao EA, Abrink M, et al. Loss of Bladder Epithelium Induced by Cytolytic Mast Cell Granules. Immunity. 2016;45(6):1258-69. Epub 2016/12/13. doi: 10.1016/j.immuni.2016.11.003. PubMed PMID: 27939674; PubMed Central PMCID: PMCPMC5177478.

3.	Miao Y, Li G, Zhang X, Xu H, Abraham SN. A TRP Channel Senses Lysosome Neutralization by Pathogens to Trigger Their Expulsion. Cell. 2015;161(6):1306-19. Epub 2015/06/02. doi: 10.1016/j.cell.2015.05.009. PubMed PMID: 26027738; PubMed Central PMCID: PMCPMC4458218.

4.	Chan CY, St John AL, Abraham SN. Mast cell interleukin-10 drives localized tolerance in chronic bladder infection. Immunity. 2013;38(2):349-59. Epub 2013/02/19. doi: 10.1016/j.immuni.2012.10.019. PubMed PMID: 23415912; PubMed Central PMCID: PMCPMC3647685.

5.	Wong SH, Zhang T, Xu Y, Subramaniam VN, Griffiths G, Hong W. Endobrevin, a novel synaptobrevin/VAMP-like protein preferentially associated with the early endosome. Mol Biol Cell. 1998;9(6):1549-63. Epub 1998/06/17. doi: 10.1091/mbc.9.6.1549. PubMed PMID: 9614193; PubMed Central PMCID: PMCPMC25382.

6.	Ambite I, Puthia M, Nagy K, Cafaro C, Nadeem A, Butler DS, et al. Molecular Basis of Acute Cystitis Reveals Susceptibility Genes and Immunotherapeutic Targets. PLoS Pathog. 2016;12(10):e1005848. Epub 2016/10/13. doi: 10.1371/journal.ppat.1005848. PubMed PMID: 27732661; PubMed Central PMCID: PMCPMC5061333.

7.	Isaacson B, Hadad T, Glasner A, Gur C, Granot Z, Bachrach G, et al. Stromal Cell-Derived Factor 1 Mediates Immune Cell Attraction upon Urinary Tract Infection. Cell Rep. 2017;20(1):40-7. Epub 2017/07/07. doi: 10.1016/j.celrep.2017.06.034. PubMed PMID: 28683322.

8.	Darville T, Andrews CW, Jr., Sikes JD, Fraley PL, Rank RG. Early local cytokine profiles in strains of mice with different outcomes from chlamydial genital tract infection. Infect Immun. 2001;69(6):3556-61. Epub 2001/05/12. doi: 10.1128/IAI.69.6.3556-3561.2001. PubMed PMID: 11349013; PubMed Central PMCID: PMCPMC98334.

9.	Hung CS, Dodson KW, Hultgren SJ. A murine model of urinary tract infection. Nat Protoc. 2009;4(8):1230-43. Epub 2009/08/01. doi: 10.1038/nprot.2009.116. PubMed PMID: 19644462; PubMed Central PMCID: PMCPMC2963178.

10.	Bowen SE, Watt CL, Murawski IJ, Gupta IR, Abraham SN. Interplay between vesicoureteric reflux and kidney infection in the development of reflux nephropathy in mice. Dis Model Mech. 2013;6(4):934-41. Epub 2013/03/23. doi: 10.1242/dmm.011650. PubMed PMID: 23519031; PubMed Central PMCID: PMCPMC3701213.

11.	O'Hanley P, Low D, Romero I, Lark D, Vosti K, Falkow S, et al. Gal-Gal binding and hemolysin phenotypes and genotypes associated with uropathogenic Escherichia coli. N Engl J Med. 1985;313(7):414-20. Epub 1985/08/15. doi: 10.1056/NEJM198508153130704. PubMed PMID: 2862582.

12.	Sandberg T, Kaijser B, Lidin-Janson G, Lincoln K, Orskov F, Orskov I, et al. Virulence of Escherichia coli in relation to host factors in women with symptomatic urinary tract infection. J Clin Microbiol. 1988;26(8):1471-6. Epub 1988/08/01. doi: 10.1128/jcm.26.8.1471-1476.1988. PubMed PMID: 3049654; PubMed Central PMCID: PMCPMC266644.

13.	Orskov I, Svanborg Eden C, Orskov F. Aerobactin production of serotyped Escherichia coli from urinary tract infections. Med Microbiol Immunol. 1988;177(1):9-14. Epub 1988/01/01. doi: 10.1007/BF00190306. PubMed PMID: 3277028.

14.	Schreiber HLt, Conover MS, Chou WC, Hibbing ME, Manson AL, Dodson KW, et al. Bacterial virulence phenotypes of Escherichia coli and host susceptibility determine risk for urinary tract infections. Sci Transl Med. 2017;9(382). Epub 2017/03/24. doi: 10.1126/scitranslmed.aaf1283. PubMed PMID: 28330863; PubMed Central PMCID: PMCPMC5653229.

15.	O'Hanley P, Lalonde G, Ji G. Alpha-hemolysin contributes to the pathogenicity of piliated digalactoside-binding Escherichia coli in the kidney: efficacy of an alpha-hemolysin vaccine in preventing renal injury in the BALB/c mouse model of pyelonephritis. Infect Immun. 1991;59(3):1153-61. Epub 1991/03/01. doi: 10.1128/iai.59.3.1153-1161.1991. PubMed PMID: 1671776; PubMed Central PMCID: PMCPMC258381.

16.	Wu J, Bao C, Reinhardt RL, Abraham SN. Local induction of bladder Th1 responses to combat urinary tract infections. Proc Natl Acad Sci U S A. 2021;118(10). Epub 2021/03/04. doi: 10.1073/pnas.2026461118. PubMed PMID: 33653961; PubMed Central PMCID: PMCPMC7958421.

17.	Wankel B, Ouyang J, Guo X, Hadjiolova K, Miller J, Liao Y, et al. Sequential and compartmentalized action of Rabs, SNAREs, and MAL in the apical delivery of fusiform vesicles in urothelial umbrella cells. Mol Biol Cell. 2016;27(10):1621-34. Epub 2016/03/25. doi: 10.1091/mbc.E15-04-0230. PubMed PMID: 27009205; PubMed Central PMCID: PMCPMC4865319.

18.	Bishop BL, Duncan MJ, Song J, Li G, Zaas D, Abraham SN. Cyclic AMP-regulated exocytosis of Escherichia coli from infected bladder epithelial cells. Nat Med. 2007;13(5):625-30. Epub 2007/04/10. doi: 10.1038/nm1572. PubMed PMID: 17417648.

19.	Saffi GT, Botelho RJ. Lysosome Fission: Planning for an Exit. Trends Cell Biol. 2019;29(8):635-46. Epub 2019/06/07. doi: 10.1016/j.tcb.2019.05.003. PubMed PMID: 31171420.

20.	Thankavel K, Madison B, Ikeda T, Malaviya R, Shah AH, Arumugam PM, et al. Localization of a domain in the FimH adhesin of Escherichia coli type 1 fimbriae capable of receptor recognition and use of a domain-specific antibody to confer protection against experimental urinary tract infection. J Clin Invest. 1997;100(5):1123-36. Epub 1997/09/01. doi: 10.1172/JCI119623. PubMed PMID: 9276729; PubMed Central PMCID: PMCPMC508287.

21.	Mehershahi KS, Abraham SN, Chen SL. Complete Genome Sequence of Uropathogenic Escherichia coli Strain CI5. Genome Announc. 2015;3(3). Epub 2015/05/30. doi: 10.1128/genomeA.00558-15. PubMed PMID: 26021932; PubMed Central PMCID: PMCPMC4447917.

22.	Abraham SN, Babu JP, Giampapa CS, Hasty DL, Simpson WA, Beachey EH. Protection against Escherichia coli-induced urinary tract infections with hybridoma antibodies directed against type 1 fimbriae or complementary D-mannose receptors. Infect Immun. 1985;48(3):625-8. Epub 1985/06/01. doi: 10.1128/iai.48.3.625-628.1985. PubMed PMID: 2860067; PubMed Central PMCID: PMCPMC261209.
