# Supplementary material for: α-Hemolysin promotes uropathogenic E. coli persistence in bladder epithelial cells via abrogating bacteria-harboring lysosome acidification
Source: PLoS Pathog. 2023 May 11;19(5):e1011388. doi: 10.1371/journal.ppat.1011388 (PMC10204954; doi:10.1371/journal.ppat.1011388)
Supplement: Attachment — Submitted filename: 2nd_Response to reviewer_Plos pathogens_Final_re.docx [file ppat.1011388.s025.docx]

**Reviewer's Responses to Questions**

**Part I - Summary**

**Reviewer #1: This is a revised version of the manuscript detailing the molecular stages during the bladder epithelial infection by UPEC. The authors have carefully answered every concern raised by me and by other reviewers. However, I still have concerns.**

**Reviewer #2: The authors have fully addressed my concerns.**

**Reviewer #4: This manuscript “α-Hemolysin promotes uropathogenic E. coli persistence in bladder epithelial cells via abrogating bacteria-harboring lysosome acidification” by Naskar et al, describes the intracellular lifecycle of UPEC. Specifically, they use cellular and molecular biology techniques to probe the vesicular compartments of the host cell to assess the location of UPEC. This study uses a variety of complimentary techniques to answer very specific questions about the stages of intracellular movement. They conclude that although initially encased in RAB27b+ vesicles, UPEC briefly escapes into the cytosol before recapture in the autophagosome. Here, UPEC can prevent acidification and maturation of the autolysosome by inhibiting host V-ATPase via microtubulin instability. From the bacterial perspective, they use UPEC strain CFT073 and an isogenic hemolysin mutant to state that the hemolysin toxin is critical for RAB27b+ vesicle escape and prevention autolysosome acidification. The ultimate conclusion is that hemolysin facilitates long-term persistence in the host bladder.**

**While several sections of this manuscript have been revised and/or improved by additional experiments, there still remain critical points highlighted during the initial review that were not adequately addressed.**

**In addition, the techniques are very robust and novel; however, there are still concerns that the conclusions drawn by the authors are not fully supported by the data presented, and several key experiments are lacking appropriate cellular controls. There must be more textual clarity and mention of the experimental limitations present in this study.**

**Part II – Major Issues: Key Experiments Required for Acceptance**

**Please use this section to detail the key new experiments or modifications of existing experiments that should be absolutely required to validate study conclusions.**

**Generally, there should be no more than 3 such required experiments or major modifications for a "Major Revision" recommendation. If more than 3 experiments are necessary to validate the study conclusions, then you are encouraged to recommend "Reject".**

**Reviewer #1: The authors mention that Figures 1A and 1C represent three and two experiments, respectively and each dot represents a single mouse. Did authors include 5 mice each in three and two replicates for Fig 1A and C? This should be clearly stated in Figure legend.**

**The authors also say "we have modified the bar graph in Figure 1A with the median as the central tendency and SD."--This change is not reflected in the figure legend. This needs to be corrected. Also, standard deviation should be removed and median should be shown as a line and not as a bar.**

**I agree with the authors that "The Mann-Whitney U test is used to compare differences between two independent groups when the dependent variable is either ordinal or continuous, but not normally distributed." Since the mouse CFU data are not normal, they should use Kruskal-Wallis test.**

🡺 We appreciate the reviewer's valuable feedback and suggestions, which have enabled us to improve the quality of our manuscript.

(1) We have revised the figure legends of **Figure 1A** and **Figure 1C** to provide more detailed descriptions.

(2) We have removed standard deviation and inserted median without bar graph (**Figure 1A**).

(3) We had incorporated a description of the error bars and statistical analysis at the end of the figure 1 legends (**Line 607**).

(3) Regarding statistical analysis, we have now employed the Kruskal-Wallis test to determine significant differences among groups as suggested by the reviewer.

**Reviewer #2: NA**

**Reviewer #4: Major Comments:**

**1. Results section “Trafficking of intracellular UPEC from RAB27b vesicles into LC3A/B+ compartments is proceeded by a brief cytosolic phase”. It is unclear if autophagic and RAB27b+ vesicles co-localize. There is no microscopy image that shows both markers simultaneously and Figure 2D does not preclude that both markers are present on the same vesicle. Since autophagy is a known protein recycling pathway, it is plausible that LC3A/B+ compartments encompass damaged (via hemolysin) RAB27b+ vesicles; therefore, there would be no true cytosolic stage for the bacteria. Because of this, the subsequent knock down of ATG5 only proves that RAB27b+ vesicles have been degraded, but not that a cytosolic stage exists when autophagy is functional. A differential centrifugation prep would be more conclusive to test for viable bacteria in the cytosolic fraction. This was a missed opportunity that could have been used in conjunction with targeted enrichment for vesicle-associated UPEC.**

Figure R1. UPEC localization in RAB27b vesicles. Human 5637 BECs were transfected with pEGFP-LC3 to specifically trace LC3 autophagy component. Next day, the cells were infected with UPEC CI5 strain. After 4 h p.i., cells were stained with anti-*E. coli* (red) and anti-RAB27b (blue) antibodies. Representative images are from randomly selected regions. Scale bars: 10 μm.

**🡺** We appreciate the reviewer's insightful comments and suggestions on our manuscript. In response to this feedback, we have conducted more experiments to address the specific concerns of this reviewer. These are as follows:

As the reviewer has questions regarding the existence of a brief cytolytic phase between UPEC trafficking from RAB27b vesicles to LC3A/B vesicles, we conducted additional experiments to further elucidate this proposed transition phase (4-8 h post-infection). Specifically, we closely examined for the presence of the LC3 component (LC3B) on UPEC containing RAB27b vesicles at 4 h post-infection (**Figure R1**). To mitigate potential non-specific antibody staining issues when probing for LC3, we utilized human 5637 BECs harboring an LC3B-expressing plasmid which will allow us to specifically monitor LC3B. Notably, we did not detect any co-localization of the LC3 component on RAB27b^+^ UPEC-containing vesicles (**Figure R1**).

Figure R2. UPEC localization in LC3 autophagosome without the presence of RAB27b molecule. Human 5637 BECs were transfected with pEGFP-LC3 to specifically trace LC3 autophagy component. Next day, the cells were infected with UPEC CI5 strain. After 8 h p.i., cells were stained with anti-*E. coli* (red) and anti-RAB27b (blue) antibodies. Representative images are from randomly selected regions. Scale bars: 10 μm.

Subsequently, when we observed the cell samples at a later time-point (8 h post-infection), there were no co-localization of RAB27b and LC3B (**Figure R2**). These findings indicate that the UPEC-containing vesicles at 8 h were mostly LC3B^+^ vesicle without any RAB2b7 co-localization (**Figure R2**), suggesting that autophagy components were not associating with RAB27b harboring UPEC at any time. We deduce that the only way this transition of UPEC from Rab27^+^ vesicles to LC3b vesicles can occur is via a cytosolic phase.

In response to the reviewer's suggestion, we attempted differential centrifugation which is a commonly used technique that exploits differences in the size, shape, and density of particles within a sample to separate them under the influence of centrifugal force. However, it appears that the density difference between UPEC and UPEC-containing vesicle membranes was too subtle to be separated using differential centrifugation. Additionally, we thoroughly searched the literature to find relevant references for this experimental procedure and for this particular purpose, but we were unable to find any.

Interestingly, when we compared the physical localization of RAB27b and LC3B vesicles at 4h and 8 h post infection, respectively, we observed that RAB27b^+^ vesicles were predominantly located at the periphery of the cell, whereas LC3B^+^ vesicles, many of them bearing UPEC, appeared localized to the perinuclear region. These observations reveal spatiotemporal localization of UPEC at 4 h and 8 h post-infection which is consistent with the fact that autophagosomes are often formed on the endoplasmic reticulum (ER) [1]. This observation suggests Rab27b^+^ vesicles and LC3b^+^ vesicles are generally not close to each other and therefore unlikely to fuse with each other. Our observation supports our idea of a cytosolic phase where recently released bacteria migrate towards the center of the cell where they are “recaptured” by the autophagy apparatus of the cell.

In summary, the spatiotemporal separation of intracellular UPEC borne in distinct vesicles supports our conclusion that UPEC escapes from peripheral RAB27b^+^ vesicles and subsequently undergoes LC3-dependent autophagosome uptake near the endoplasmic reticulum.

The above data and description have been added to the results section. This information is included in the manuscript (**Line 133**) and in the figures (**S4B** and **S4C Fig**).

**The field uses 5637 cells; however, it must be mentioned somewhere (in addition to the statements in lines 408-411) the considerations of these cells being of cancerous origin. It is known that many cellular pathways (including autophagy) function differently in cancer cells.**

🡺 We have incorporated this information into the Results section (**Line 111**).

**2. Results section “HlyA facilitates the escape of UPEC from RAB27b+ vesicles”**

**To support this claim, authors must include the genetic complementation of CFT in these specific, keystone experiments. Ideally, there would be similar studies in a HlyA- UPEC strain as a control to demonstrate that these phenomena are truly hemolysin-mediated.**

Figure R3. Escape of *ΔhlyD* UPECs from RAB27b vesicles after complementation. *ΔhlyD* UPEC strain was transformed with *phlyD* plasmid to generate *ΔhlyD*(*phlyD*) strain. Human 5637 BECs were infected with magnetic beads labeled *ΔhlyD*(*phlyD*) strain or *ΔhlyD* with empty plasmid. After 5 h of infection, isolated UPEC-containing vesicles were analyzed for the expression of RAB27b. Gapdh was used for loading control of UPEC-containing vesicles.

🡺 We performed a complementation study using a *ΔhlyD* strain that was transformed with either a phlyD plasmid or an empty plasmid. We subsequently infected human BECs with equal numbers of *ΔhlyD*(*phlyD*) or control *ΔhlyD* strain and compared their ability to escape from RAB27b^+^ vesicles. Notably, at 5 h post-infection, the *ΔhlyD*(*phlyD*) strain exhibited lower RAB27b marker expression on western blot data compared to the control *ΔhlyD* strain expressing the empty plasmid (**Figure R3**). Thus, these results support the notion that hemolysin facilitates the escape of UPEC from RAB27b^+^ vesicles. The aforementioned findings have been added to the results section.

This information is included in the manuscript (**Line 202**) and has been added in the figure (**S12 Fig**).

**The cell monolayer would be partially permeable or “leaky” (even though sublytic) with many of these assays due to hemolysin insertion into the plasma membrane. At what point does it become gentamicin sensitive in this process? Similarly, the RAB27b+ vesicle would also go through various stages of degradation via hemolysin.**

🡺 We appreciate the critical inquiry regarding the distinction between HlyA-mediated effects on plasma membrane versus vesicular membrane. While both membranes are composed of lipid bilayers, the biochemical and biophysical response of these membranes to HlyA on these membranes are likely to be different. Specifically, the plasma membrane typically has a higher concentration of cholesterol than the vesicular membrane, which is rich in phosphatidylserine concentration [2]. Moreover, due to their small size and spherical shape, the vesicular membrane often exhibits a higher degree of curvature than the plasma membrane, as the packing of lipids in curved regions can be different from that in flat regions [2]. Lastly, plasma membrane proteins are replete with ion channels, transporters, and receptor proteins, whereas vesicular membrane is replete with proteins associated with vesicle fusion, cargo sorting, and the regulation of vesicle formation [3]. These differences in the membrane-embedded proteins between vesicular and plasma membranes may result in differing outcomes when exposed to HlyA. Taken together, the differences described above suggest that HlyA-mediated perturbations of the plasma membrane is likely to be distinct from those of the vesicular membrane.

We do not think that HlyA insertion into the plasma membrane causes BECs to become partially permeable to extracellular gentamycin during UPEC infection while still remaining viable. Our reading of the literature suggests that it is difficult for HlyA to stably form pores without lysing cells, except perhaps during the reconstitution of HlyA channels on artificial lipid bilayer membranes [4]. Furthermore, a recent study demonstrated that HlyA at high concentrations is capable of rapid lytic cell death during UPEC infection. HlyA apparently, activates Caspase 1 and 4 [5]. Therefore, if BECs are susceptible to gentamicin during UPEC infection, it would have to be after HlyA-dependent lytic cell death.

Along those lines, it is notable that host cells infected with *Listeria monocytogenes* which produce ample amounts of a hemolysin, Listeriolysin O, do not undergo sudden lytic cell death but instead harbor a cytosolic stage of *L. monocytogenes* for significant periods of time without the rupture of the host plasma membrane. While the hemolysin of UPEC is a critical factor in causing lytic plasma membrane, host membrane homeostasis is also a crucial factor in determining when membrane lysis is initiated.

In summary, the biological differences between plasma membrane and vesicular membrane contribute to their distinct susceptibility to HlyA-mediated lysis.

This information is included in the manuscript (**Line 419**).

**Why the switch from MOI 50 to 20?**

🡺 In Figure 3E, an MOI of 20 was utilized to ensure a sublytic condition of UPEC infection and to prevent plasma membrane lysis.

**The data in Figure 3G are highly variable and are not truly indicative of expulsion (rather than IBC formation followed by cell death) without more data. The clusters of bacteria are visible in most of the microscopy images. QIRs have been described as single bacteria in a vesicle. IBC are large clusters that grow in the cytoplasm until the cell bursts. How do these fit into the paradigm? Although there is brief mention, more explanation is needed.**

🡺 We acknowledge the reviewer's critique. Accordingly, we have removed the following italicized sentence from result section (**Line 207**). *“Presumably, when encased in RAB27b compartments, the ΔhlyD strain was expelled by BECs, whereas the CFT073 strain was not; this deduction was confirmed”*.

Previous studies conducted by different research groups have observed the presence of UPEC clusters within individual vesicles of human 5637 BECs or mouse bladder superficial epithelial cells [6, 7]. Thus, our observation of UPEC clusters within LAMP1 vesicles is consistent with the findings of other research groups. Presumably the UPEC within the LAMP1 vesicles that we observed in our could be closer to quiescent bacteria rather than intracellular bacterial communities (IBCs) with large clusters that grow rapidly in the cytoplasm. It is notable that intracellular UPEC numbers remain stable over 120 h post-infection (**Figure 1E**) while these UPECs are still detectable in LAMP1 vesicles (**Figure 4E**). Thus, it is not easy for us to readily associate our phenotype to quiescent intracellular residence (QIR) or to cytosolic IBC or how and when our phenotype transitions to these well described intracellular states. Interestingly, another research group has provided valuable insights into the transition mechanism from QIR to IBC which appears distinctively different from transition that we have described in our study. This study demonstrated that treatment of UPEC-harboring host cells with saponin led to a rapid and substantial increase in total intra- and extracellular bacterial numbers, with the formation of intracellular, pod-like bacterial inclusions [7]. This result suggests that the transition from QIR to IBC formation may require environmental stimuli, such as reactive oxygen stress from neutrophils, alarmins from dying neighboring host cells, sudden fluctuations of cytosolic ions from the activation of ion channels, or dysbiosis of the bladder tissue microenvironment.

This information is included in the manuscript (**Line 396**).

**3. Results section “Inhibition of V-ATPase recruitment by HlyA” and “Destabilization of the cytoskeletal structure in bacteria-infected BECs reduces intracellular bacterial killing”**

**The narrative mentions expression of hemolysin while the UPEC are intracellular; however, the experimental design does not test this directly. This is critical due to the verbiage in this section and in the discussion. The vesicle isolation preps could have been blotted for HlyA and/or a luminescent reporter containing the hlyA promoter could have been used.**

🡺 We have found providing experimental evidence for intracellular bacterial secretion of HlyA challenging. We suspect this is because the hly-operon is critically regulated by the growth phase [8]. In the phase of growth, they are in within the intracellular compartments, the amounts of HlyA produced appear to be too small to detect using standard approaches. In any case, we wish to highlight a study by others utilizing hemolysin overexpression techniques. These studies revealed intracellularly produced hemolysin caused the rupture of intracellular vacuoles housing bacteria in host cells, resulting in HlyA release into the cytosol. At this cytosolic site, HlyA triggered caspase-11/4-mediated noncanonical inflammasome activation [9]. These studies directly support bacterial production of HlyA intracellularly and its role in lysing vesicle membrane.

The above description has been added to the discussion section. This information is included in the manuscript (**Line 375**).

**We can’t be sure that the hlyD mutant doesn’t have altered regulation of other virulence factors preventing acidification. These experiments must be performed with the complemented mutant to support these statements.**

🡺 We have now complemented the *ΔhlyD* strain with either the *phlyD* plasmid or the empty plasmid and infected 5637 BECs. After 24 h post-infection, we examined the acidification of UPEC-containing LAMP1 vesicles (**Figure R4**). The *ΔhlyD* strain with the empty plasmid consistently exhibited acidification in LAMP1 vesicles; however, the *ΔhlyD*(*phlyD*) strain lacked this acidification (**Figure R4**). Quantification of the level of acidification in UPEC-containing vesicles revealed a significant reduction when the *ΔhlyD* strain was complemented with the *phlyD* plasmid.

The aforementioned findings have been added to the results section. This information is included in the manuscript (**Line 238**) and has been added in the figure (**S16 Fig**).

Figure R4. Deacidification of *ΔhlyD* UPECs after complementation of *hlyD* gene. *ΔhlyD* UPEC strain was transformed with *phlyD* plasmid to generate *ΔhlyD*(*phlyD*) strain. Human 5637 BECs were infected with *ΔhlyD*(*phlyD*) strain or *ΔhlyD* with empty plasmid. After 24 h of infection, BECs were dyed with LysoTracker (red) to trace the acidification of UPEC-containing vesicles. Anti-UPEC (green) and anti-LAMP1 (blue) antibodies were used to stain the fixed cells. Quantitative data from two independent experiments were analyzed. Data are shown as mean ±SD. Data were analyzed by unpaired two-tailed Student’s *t*-test. ****P<0.0001. Scale bar: 10 μm.

**The wording makes it seem as if HlyA has a direct interaction with V-ATPase at this particular stage of the intracellular lifecycle. Could it not be the hemolysin inserted into the plasma membrane that is destabilizing the cytoskeleton and thus V-ATPase recruitment? The findings in Fig. 5A demonstrate that the cell architecture has been compromised. It could be from the effects of hemolysin secreted extracellularly leading to microtubule dysregulation inside the cell.**

🡺 In view of the reviewer comment, regarding the possible effects of extracellularly secreted HlyA on microtubule dysregulation we have modified the text in the manuscript accordingly (**Line 385**).

Figure R5. Isolated magnetic bead-labeled UPEC associated with caveolin-1. Human 5637 BECs were incubated with magnetic beads alone or with magnetic bead-labeled UPEC. After 2 h of incubation, isolated beads were analyzed for the expression of Caveolin-1.

**With many of these experiments, plasma membrane was selectively disrupted when it could have been isolated and/or stained as a negative internal control. Couldn’t the gentamicin-sensitive UPEC be associated with the membrane? A co-IP for plasma membrane associated protein (clathrin/calveolin/dynamin) could address this.**

🡺 To address the concerns regarding non-specific interactions of magnetic bead associated UPEC with host cellular components or signaling molecules, we examined the presence of endobrevin in UPEC-containing vesicles (**Figure S6**). Additionally, to confirm the technical robustness of our methodology, we performed western blot analysis of Caveolin-1 in the same samples treated with either magnetic beads bound UPEC or magnetic beads alone. Interestingly, as shown in **Figure R5**, we observed the presence of Caveolin-1 in both conditions at 2 h post-infection or post-treatment. However, we believe that the presence of Caveolin-1 in UPEC-containing vesicles is not due to non-specific interactions between the plasma membrane and vesicle membrane, but rather the consequence of magnetic beads and magnetic UPEC being internalized by host cells through lipid rafts [10]. This association between UPEC-containing vesicles and Caveolin-1 disappeared when we compared the expression of Caveolin-1 at 2 h and 24 h post-infection (**Figure R6**). This result not only supports the robustness and consistency of our analysis but also suggests that UPEC-containing exocytic vesicles in the early stage of infection have distinct characteristics compared to LAMP1^+^ vesicles in the late stage of UPEC infection. Furthermore, this result provides evidence for a change in the integrity of vesicles containing UPEC from plasma membrane-derived vesicles to cytosolic component-derived vesicles. This finding supports our earlier claim that UPEC escapes from fusiform vesicles and undergoes a transition into autophagosome and autolysosome maturation.

Figure R6. Differential association of UPEC-containing vesicles with Caveloin-1. Human 5637 BECs were infected with magnetic bead- labeled UPEC for 2 or 24 h. Isolated UPEC-containing vesicles were analyzed for the presence of Caveolin-1. Gapdh was used for loading control of UPEC-containing vesicles.

**4. Based on the fact that only 30-50% (as stated in the introduction) of UPEC are hlyA+, some of the statements about future therapeutics are not fully supported by these data.**

🡺 We agree with the reviewer. As a result, we revised the sentence regarding the therapeutic emphasis of the paclitaxel application (**Line 441**).

**Part III – Minor Issues: Editorial and Data Presentation Modifications**

**Please use this section for editorial suggestions as well as relatively minor modifications of existing data that would enhance clarity.**

**Reviewer #1: In general, there are a lot of changes in figures although they are not always reflected in the figure legend. At least they are highlighted in yellow.**

**Include limit of detection in ALL figures showing CFU/ml data-Fig 1A, 1C, Fig 6C, etc. Explain in methods how the LOD was determined.**

🡺 We apologize the unclear description of LOD in the previous version of manuscript. Here, we included the supplemental figures in which **Fig1A**, **Fig6A**, and **Fig6C** were converted into CFU/mL. We also described the limit of detection in the legend of this figure and the material and method section (**S22 Fig** and **Line 462**).

**Reviewer #2: The authors have fully addressed my concerns**

**Reviewer #4: Minor Comments:**

**• I assume CI5 is hlyA+, but it is not explicitly stated**

🡺 The reviewer is correct. Our hemolysis analysis (**Figure 3A**) along with complete genome sequencing confirmed hlyA^+^. The description was added at **Line 444**.

**• Add Fig S1 to panel Fig 1A**

🡺 We have decided to keep **Figure S1** separate from **Figure 1A** because the experiment shown in this particular panel was not performed with the same set of samples as **Figure 1A**. Moreover, as the experimental dates of **Figure 1A** were synchronized with those in **Figures 1B** and **Figure 1C**, we believe that keeping this panel as a separate figure is more consistent with the study and more acceptable to the reader.

**• Line 103-104: replace with “neutrophil recruitment had subsided”**

🡺 We revised the sentence as the reviewer suggested (**Line 102**).

**• rUTI patient samples during an episode of UTI or during “dormancy”?**

🡺 The samples were obtained from patients who tested positive for the presence of uropathogens.

**• What is the genotype (hlyA+ or -) of rUTI strains shown here?**

🡺 *E. coli* CFT073 was reported to have hly^+^ pap^+^ sfa^+^ pil^+^ strain [11]. A hlyA- isogenic mutant, *ΔhlyD* strain, is *E. coli* CFT073 hlyD::TnphoA [11].

**• Were gentamicin concentrations tested on CI5 to determine sensitivity?**

🡺 We validated the sensitivity of CI5 strain to gentamicin using a specific concentration. The experimental protocol was previously established in a published study [12, 13].

**• Why include J96 (fig. 3A) if there is no mention in the text?**

**🡺** We included the description in the text (**Line 184**).

**• Line 114: What is this number as a percent of total UPEC infected? (only 0.01% of UPEC enters the cell at MOI 100)**

🡺 The reviewer is right. An approximate value of 0.01% was obtained when calculating the intracellular UPEC number at 12 h post-infection relative to the initial UPEC number (**Figure 1E**).

**• Triton-X should have been an included positive control in cytotoxicity assays**

🡺 The treatment of Triton-X100 was included in the cytotoxicity assay because “Lysis solution” in the assay kit (Promega, Cytotox 96 Non-radioactive assay kit) to measure the maximum LDH release from cells already contains 8% Triton X-100. Therefore, the readout of the Triton-X100 treated wells (Lysis solution treated condition) served as a positive control for maximum LDH release.

We observed that the readout of the Triton-X100 treated wells (Lysis solution treated condition) was consistently higher than that of the experimental wells with UPEC infection.

**• Clusters of UPEC in autolysosomes (as seen in microscopy images) would skew these CFU data**

🡺 We agree the reviewer’s concern. Although clusters of UPEC in autolysosomes or LAMP1^+^ vesicles were detected through microscopic images, the actual colony-forming unit (CFU) counts of intracellular UPEC in BECs remained at similar levels from 24 to 120 hours post-infection (**Figure 1E**). Therefore, our overall conclusion about the presence of persistent UPEC in these vesicles remains unaffected.

**• Figure 6B can’t be 0 CFU unless the entire organ homogenate was plated, which is not the case according to the methods**

🡺 We agree with the reviewer's suggestion, and as a result, we have replaced the value of 0 with ND (not detectable). Please see our comment on minor issue #1 of Reviewer #1.

**• Gentamicin misspelled in figures**

🡺 We corrected typos.

**• Does Fig. S20 indicate that there is only n=1 for WB experiments**

🡺 Representative results from two to three independent Western blots were selected and presented.

**References**

1.	Nakatogawa H. Mechanisms governing autophagosome biogenesis. Nat Rev Mol Cell Biol. 2020;21(8):439-58. Epub 2020/05/07. doi: 10.1038/s41580-020-0241-0. PubMed PMID: 32372019.

2.	van Meer G, Voelker DR, Feigenson GW. Membrane lipids: where they are and how they behave. Nat Rev Mol Cell Biol. 2008;9(2):112-24. Epub 2008/01/25. doi: 10.1038/nrm2330. PubMed PMID: 18216768; PubMed Central PMCID: PMCPMC2642958.

3.	Bonifacino JS, Glick BS. The mechanisms of vesicle budding and fusion. Cell. 2004;116(2):153-66. Epub 2004/01/28. doi: 10.1016/s0092-8674(03)01079-1. PubMed PMID: 14744428.

4.	Glazier SA, Vanderah DJ, Plant AL, Bayley H, Valincius G, Kasianowicz JJ. Reconstitution of the Pore-Forming Toxin α-Hemolysin in Phospholipid/18-Octadecyl-1-thiahexa(ethylene oxide) and Phospholipid/n-Octadecanethiol Supported Bilayer Membranes. Langmuir. 2000;16(26):10428-35. doi: 10.1021/la000690k.

5.	Nagamatsu K, Hannan TJ, Guest RL, Kostakioti M, Hadjifrangiskou M, Binkley J, et al. Dysregulation of Escherichia coli alpha-hemolysin expression alters the course of acute and persistent urinary tract infection. Proc Natl Acad Sci U S A. 2015;112(8):E871-80. Epub 2015/02/13. doi: 10.1073/pnas.1500374112. PubMed PMID: 25675528; PubMed Central PMCID: PMCPMC4345586.

6.	Justice SS, Hung C, Theriot JA, Fletcher DA, Anderson GG, Footer MJ, et al. Differentiation and developmental pathways of uropathogenic Escherichia coli in urinary tract pathogenesis. Proc Natl Acad Sci U S A. 2004;101(5):1333-8. Epub 2004/01/24. doi: 10.1073/pnas.0308125100. PubMed PMID: 14739341; PubMed Central PMCID: PMCPMC337053.

7.	Eto DS, Sundsbak JL, Mulvey MA. Actin-gated intracellular growth and resurgence of uropathogenic Escherichia coli. Cell Microbiol. 2006;8(4):704-17. Epub 2006/03/22. doi: 10.1111/j.1462-5822.2006.00691.x. PubMed PMID: 16548895.

8.	Nicaud JM, Mackman N, Gray L, Holland IB. Regulation of haemolysin synthesis in E. coli determined by HLY genes of human origin. Mol Gen Genet. 1985;199(1):111-6. Epub 1985/01/01. doi: 10.1007/BF00327519. PubMed PMID: 3923298.

9.	Chen S, Yang D, Wen Y, Jiang Z, Zhang L, Jiang J, et al. Dysregulated hemolysin liberates bacterial outer membrane vesicles for cytosolic lipopolysaccharide sensing. PLoS Pathog. 2018;14(8):e1007240. Epub 2018/08/24. doi: 10.1371/journal.ppat.1007240. PubMed PMID: 30138458; PubMed Central PMCID: PMCPMC6124777.

10.	Shin JS, Gao Z, Abraham SN. Involvement of cellular caveolae in bacterial entry into mast cells. Science. 2000;289(5480):785-8. Epub 2000/08/05. doi: 10.1126/science.289.5480.785. PubMed PMID: 10926542.

11.	Mobley HL, Green DM, Trifillis AL, Johnson DE, Chippendale GR, Lockatell CV, et al. Pyelonephritogenic Escherichia coli and killing of cultured human renal proximal tubular epithelial cells: role of hemolysin in some strains. Infect Immun. 1990;58(5):1281-9. Epub 1990/05/01. doi: 10.1128/iai.58.5.1281-1289.1990. PubMed PMID: 2182540; PubMed Central PMCID: PMCPMC258621.

12.	Song CH, Kim YH, Naskar M, Hayes BW, Abraham MA, Noh JH, et al. Lactobacillus crispatus Limits Bladder Uropathogenic E. coli Infection by Triggering a Host Type I Interferon Response. Proc Natl Acad Sci U S A. 2022;119(33):e2117904119. Epub 2022/08/09. doi: 10.1073/pnas.2117904119. PubMed PMID: 35939684; PubMed Central PMCID: PMCPMC9388105.

13.	Choi HW, Bowen SE, Miao Y, Chan CY, Miao EA, Abrink M, et al. Loss of Bladder Epithelium Induced by Cytolytic Mast Cell Granules. Immunity. 2016;45(6):1258-69. Epub 2016/12/13. doi: 10.1016/j.immuni.2016.11.003. PubMed PMID: 27939674; PubMed Central PMCID: PMCPMC5177478.
